# Quantification of Pancreatic Cancer Proteome and Phosphorylome: Indicates Molecular Events Likely Contributing to Cancer and Activity of Drug Targets

**DOI:** 10.1371/journal.pone.0090948

**Published:** 2014-03-26

**Authors:** David Britton, Yoh Zen, Alberto Quaglia, Stefan Selzer, Vikram Mitra, Christopher Lößner, Stephan Jung, Gitte Böhm, Peter Schmid, Petra Prefot, Claudia Hoehle, Sasa Koncarevic, Julia Gee, Robert Nicholson, Malcolm Ward, Leandro Castellano, Justin Stebbing, Hans Dieter Zucht, Debashis Sarker, Nigel Heaton, Ian Pike

**Affiliations:** 1 Proteome Sciences plc, Cobham, United Kingdom; 2 Institute of Liver Studies, King's College Hospital, London, United Kingdom; 3 Faculty of Medicine, Department of Surgery & Cancer, Imperial College, London, United Kingdom; 4 Cardiff School of Pharmacy & Pharmaceutical Sciences, Cardiff University, Cardiff, United Kingdom; University of Iowa, United States of America

## Abstract

**Objective:**

LC-MS/MS phospho-proteomics is an essential technology to help unravel the complex molecular events that lead to and propagate cancer. We have developed a global phospho-proteomic workflow to determine activity of signaling pathways and drug targets in pancreatic cancer tissue for clinical application.

**Methods:**

Peptides resulting from tryptic digestion of proteins extracted from frozen tissue of pancreatic ductal adenocarcinoma and background pancreas (n = 12), were labelled with tandem mass tags (TMT 8-plex), separated by strong cation exchange chromatography, then were analysed by LC-MS/MS directly or first enriched for phosphopeptides using IMAC and TiO_2_, prior to analysis. In-house, commercial and freeware bioinformatic platforms were used to identify relevant biological events from the complex dataset.

**Results:**

Of 2,101 proteins identified, 152 demonstrated significant difference in abundance between tumor and non-tumor tissue. They included proteins that are known to be up-regulated in pancreatic cancer (e.g. Mucin-1), but the majority were new candidate markers such as HIPK1 & MLCK. Of the 6,543 unique phosphopeptides identified (6,284 unique phosphorylation sites), 635 showed significant regulation, particularly those from proteins involved in cell migration (Rho guanine nucleotide exchange factors & MRCKα) and formation of focal adhesions. Activator phosphorylation sites on FYN, AKT1, ERK2, HDAC1 and other drug targets were found to be highly modulated (≥2 fold) in different cases highlighting their predictive power.

**Conclusion:**

Here we provided critical information enabling us to identify the common and unique molecular events likely contributing to cancer in each case. Such information may be used to help predict more bespoke therapy suitable for an individual case.

## Introduction

Protein phosphorylation is a common process modulating the activity of oncogenic and tumor suppressor proteins [1]–[3]. In many cases, phosphorylation results in switch-like changes in protein function, due to modulation of protein folding, substrate affinity, stability, and activity of its substrates, in turn affecting signaling pathways controlling cell proliferation, migration, differentiation, and apoptosis, dysregulation of which contribute to the cancer phenotype [4]. Pancreatic cancer is one of the most aggressive malignant neoplasms with a median survival of 6 months. A significant proportion of patients are diagnosed at an advanced stage where therapy options are very limited [5]. As is the case for other cancers, molecular targeting therapy is promising for treatment of advanced or recurrent pancreatic cancer [6]. Although a variety of molecular targeting drugs have been available in the last decade and many others are also expected in the next few years, a breakthrough is still required for prediction of drug effects and drug selection. For example, sorafenib, a multi-kinase inhibitor acting on hyperactive vascular endothelial growth factor receptor, platelet-derived growth factor receptor and Raf, has proven efficacy in some patients with advanced hepatocellular carcinoma [7], but we cannot currently predict its effect on an individual patient before starting treatment. To overcome these difficulties, it seems crucial to establish an analytical approach to help drug selection, where expression and activity of multiple drug targets are comprehensively assessed on a case-by-case basis. Phosphorylation is a key event modulating protein activity, therefore measuring protein phosphorylation is a useful indicator of activation status.

There are hundreds of anti-cancer drug targets and oncogenic signaling proteins that are relevant to therapeutic selection therefore measuring expression and activation status of all using the current gold standard analysis, immunohistochemistry (IHC), is not feasible. In this respect, IHC maintains a role as a validation tool. Reverse phase protein microarrays (RPMA) have limitations due to a limited antibody repertoire and poor specificity/cross reactivity. In addition, genomics-based technologies do not allow phospho-signaling measurements. Liquid chromatography - tandem mass spectrometry (LC-MS/MS) based proteomic approaches have been developed to identify and quantify thousands of proteins and their phosphorylation sites [8], [9]. In this study we have developed an LC-MS/MS based phospho-proteomic workflow (SysQuant) to overcome many of the technical and bio-informatic difficulties involved in effectively quantifying expression and activity of signaling proteins, many of which are drug targets, at a global or system wide level in tumor tissue. We compared frozen resected tissue (tumor versus non-tumor background) from twelve cases of pancreatic head ductal adenocarcinoma and increased throughput utilising reporter ion isotopologues of TMT, resulting in 8-plex reagents and therefore the ability to run eight samples simultaneously [10], [11]. Molecular events likely to contribute to cancer were identified common to all cases however some were unique to an individual case or subgroup. There also appeared to be a relationship between time of recurrence and the grouping of cases following principal component analysis of the T/NT ratios of phosphopeptides. Phosphopeptide analysis using SysQuant may identify new therapeutic targets and also help stratify patients into different treatment regimens based on the activation status of signaling pathways and known drug targets.

## Materials and Methods

Ethical aspects and research protocol were approved by the BioBank Committee of the Institute of Liver Studies, King's College Hospital (Reference No. 08/H0704/117). All participants provided written informed consent to use their tissue samples for research. Twelve cases of pancreatic head ductal adenocarcinoma were selected (Table S1 in Tables S1). Additional non-confidential clinical information such as tumor stage, gender and recurrence can be seen for each case in Tables S2 & S3 in Tables S1. Tumor (T) tissue samples were taken from the pancreatic tumor masses, while non-tumor (NT) samples were from the pancreas away from the tumor mass. All tissue samples were frozen within 30 minutes of surgical resection and stored [at −80°C] until analysis by SysQuant (median time of storage [18.5 months] range [4–28 months]. T versus NT were compared using SysQuant and experimental details are described in the Methods S1 document. In summary, this entailed protein extraction from tissue specimens (µg amounts used for each specimen are shown in Table S4 in Tables S1), trypsin digestion of proteins into peptides, TMT 8-plex labelling of peptides (tumor and non-tumor tissue from 4 cases per TMT 8-plex) followed by mixing to form a single 8-plex sample mixture (see Table S5, in Tables S1). Each TMT 8-plex sample was then split into three independent aliquots, each of which was further split into 12 fractions by strong cation exchange (SCX) chromatography (Table S6, in Tables S1). The first set of 12 SCX fractions were then analysed directly by LC-MS/MS using duplicate data dependent acquisition runs followed by a third run using time dependent rejection of all features identified in runs 1 & 2. The remaining two sets of 12 fractions were first enriched for phosphopeptides using either immobilised metal affinity chromatography (IMAC) or TiO_2_ (Table S6, in Tables S1). The resulting 24 phosphopeptide enriched fractions were also analysed by LC-MS/MS. In total 108 separate LC-MS/MS runs were performed for each TMT 8-plex sample. Raw mass spectrometry data were searched against the human UniProtKB/Swiss-Prot database using Mascot and Sequest (via Proteome Discoverer). Peptide spectrum matches (PSMs) were rejected if identified with only low confidence (≥5% FDR), showed ≤75% phospho-RS probability score, and had missing quantification channels (e.g. not all peaks for isobaric tags visible in spectra). Raw intensity values of isobaric tags from PSMs passing filters were used for quantification, but first normalised using sum-scaling (as shown in Figure S1) to reduce potential experimental/systematic bias. Log_2_ ratios were calculated from isobaric tag intensities, showing the regulation between T over NT for each case. A phosphopeptide T/NT log_2_ ratio is the median T/NT log_2_ ratio from all PSMs unique to that specific peptide sequence. A protein T/NT log_2_ ratio is the median T/NT log_2_ ratio from all unique non-phosphorylated peptides unique to that specific protein. One sided t-test (one-sample location test) was used to calculate p-values. P-values were plotted against log_2_ T/NT ratios on Volcano plots to identify significantly regulated peptides. At the protein level, annotation using GO-terms, KEGG-pathways and Drugbank information were added, and proteins were also mapped to pathways using resources such as DAVID and STRING. At the phosphorylation site level annotation using PhosphoSitePlus were added, including known functional and biological/pathological role of the phosphorylation site. Partial Least Squares Discriminant Analysis (PLS-DA) was used to model and investigate the multivariate dataset to identify outliers and groups from all peptide isobaric tag intensities from each filter passing PSM, as well as log_2_ T/NT ratios (phosphopeptides) from all arms of the workflow (IMAC, TiO_2_ and non-enriched). The SysQuant workflow, combining phospho-proteomic sample preparation, LC-MS/MS analysis, and bioinformatics analysis, was used to identify important molecular events we believe contribute to pancreatic cancer in the cases analysed here.

## Results and Discussion

All peptides identified by Sequest and Mascot in this study were exported from Proteome Discoverer and can be viewed on the zip Files S1, S2, and S3. File S1 contains all peptides (phosphorylated and non-phosphorylated) identified from the specimens in TMT 8-plex-1, File S2 contains all peptides identified from specimens in TMT 8-plex-2, and File S3 contains all peptides identified from specimens in TMT 8-plex-3. These Supplemental zip Files display detailed information including Sequest Xcorr, Mascot ions scores, ΔM [ppm], Percolator q-values, and other important information. Data from these excel documents were input into in-house bioinformatic tools to identify biologically relevant events.

**Figure 1:**
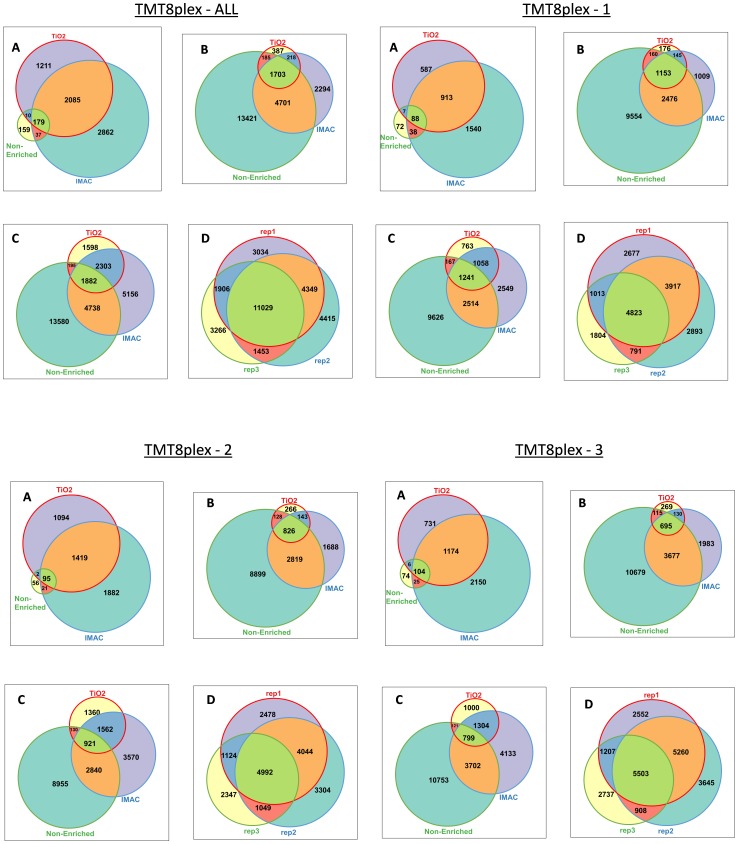
Number of Identified Peptides. Venn diagrams demonstrate the number of; A: unique phosphopeptide sequences, B: unique non-phosphopeptide sequences, and C: total number of unique peptide sequences identified in the TiO_2_, IMAC, and/or non-enrich arm of the SysQuant workflow, across all three TMT 8-plex samples in total (TMT 8-plex-ALL) and individually per TMT 8-plex (TMT 8-plex 1, TMT 8-plex 2, TMT 8-plex 3). D: demonstrates the level of overlap we observe for peptide identifications from analytical run 1, analytical run 2, and analytical run 3 (including time dependent rejection list compiled from identifications from run 1 and 2).

In total we identified 6,543 unique phosphopeptides sequences (6,284 unique phosphorylation sites), from 2,101 proteins (Table 1). Figure 1 shows identified peptide (phosphorylated and non-phosphorylated) distribution over all three arms (Non-enriched, TiO_2_, IMAC) of the SysQuant workflow for each TMT 8-plex. Figure 1 also illustrates the number of peptides detected in total for all three analytical repeats (after combining numbers from different fractions) in each and all TMT 8-plex samples. When results from each of the parallel components (TiO_2_, IMAC, non-enriched) are compared the benefits of a combined enrichment approach and multiple analytical repeats (including utilisation of the time dependent rejection list), are apparent. The largest total number of phospho-peptides was seen using IMAC enrichment which accounted for 79% of all unique phosphopeptides identified. However, the TiO_2_ fractions uniquely identified nearly 19% of the total which would be missed using a single phospho-peptide enrichment strategy (Figure 1: TMT 8-plex-ALL: A). The same is true for the three analytical runs performed on each sample. If a single data dependent run was performed only 20,318 unique peptides are seen (Figure 1: TMT 8-plex-ALL: D). A second data-dependent run adds 5,868 peptides whilst the use of the time dependent rejection list in run 3 allowed a further 3257 peptides to be identified overall. Collectively (run 2&3) this represents an additional 45% over run 1 alone and 31% of the total number of unique peptides. Importantly the peptides identified in the third run are generally of lower abundance. We also illustrate (Figure S2) the number of unique phosphopeptides and non-phosphopeptides identified in each raw file, from each SCX fraction, in each arm of the workflow (non-enrich, TiO_2_, and IMAC), from each TMT 8-plex sample (TMT 8-plex-1, 2, & 3).

**Table 1:** Number of peptide spectrum matches, number of unique peptide sequences, and number of phosphorylation sites identified in each TMT 8-plex and in total.

	# PSM (phos)	# PSM (non-phos)	# PSM (phos + non-phos)	# Unique peptides (phos)	# unique peptides (Non-phos)	# unique peptides (phos + non-phos)	# phospho-sites Mascot + Sequest
Σ TMT 8-plex −1	21428	88911	110339	3245	14673	17918	3161
Σ TMT 8-plex −2	29300	88568	117868	4569	14769	19338	4426
Σ TMT 8-plex −3	25914	102303	128217	4264	17548	21812	4184
Σ TMT 8-plex 1+2+3	**76642**	**279782**	**356424**	**6543**	**22909**	**29452**	**6284**

Of the 6543 phosphopeptides identified, 5409 were quantifiable. Due to the large number of quantifiable phosphopeptides these must be viewed on a separate excel file (File S4), rather than as part of the main document. File S4 displays the phosphopeptide sequences, the phosphorylated residues and the protein name and Uniprot accession number to which the peptide belongs. File S4 also displays all quantitative and statistical information relating to the phosphopeptides in tumor versus non-tumor from all cases, and also gives annotation information including known functional effects of the phosphorylation event. This information was extracted from the PhosphositePlus database and can be observed in columns BM-CP. File S4 also provides functional information relating to the protein, information extracted from GO terms (columns CQ-DC) and whether such proteins are known drug targets (columns DD-DG) extracted from the Drug Bank database. For additional information regarding the relative protein abundance and normalised phosphopeptide levels (phosphopeptide normalised to protein level) refer to File S5. The relative abundance of phosphopeptides in tumor versus non-tumor tissue will change from case to case primarily due to changes in expression level of the phosphorylated protein or due to modulated activity of the kinases and phosphatases inducing or reversing phosphorylation of the protein substrate, respectively. In File S5 we normalise the relative abundance of a phosphopeptide to the relative abundance of the respective protein. Relative protein abundance is calculated using only non-phosphorylated peptides therefore there are cases where we are not able to carry out normalisation due to the absence of non-phosphorylated peptides to some of the proteins.

### PLS-DA

**Figure 2:**
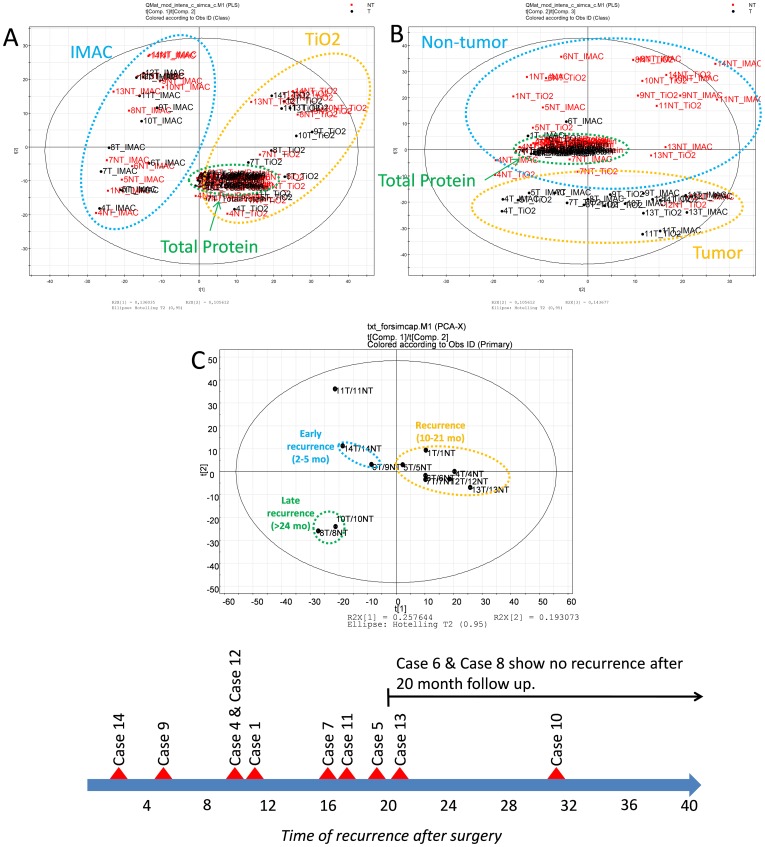
Partial Least Squares Discriminant Analysis (PLS-DA). A: PC1 and PC2 score plot of the first two principal components describing 13.6% (PC1) and 10.6% (PC2) of the total variance in the data (raw isobaric tag intensities from each PSM passing set filters). The circle depicts the T2 hotelling space based on 95% confidence. B: PC2 and PC3 score plot of the next principal components describing 10.6% (PC2) and 14.4% (PC3) of the total variance in the data. C: PC1 and PC2 score plot of the first two principal components describing 25.8% (PC1) and 19.3% (PC2) of the total variance in the data (median log_2_ T/NT ratios of all quantifiable phosphopeptides in each case). Here we also display the time of recurrence in months for each case, following surgery.

The first Principal Component (PC1) shows the variability introduced due to the three different arms of the workflow. These three arms IMAC, TiO_2_ and Total Protein (i.e non-enriched), as shown in Figure 2A and Figure S3, have separated the variables into 3 separate clusters. The solid black circle in Figure 2A depicts the T2 hotelling space based on 95% confidence. PC1 explains 13.6% of the total variance in the dataset. The second Principal Component (PC2) illustrates the variability introduced by different TMT 8-plex channels. This variability highlights primarily the patient to patient variance, which is 10.56% of the total variance in the dataset. The between class variation, i.e Tumor (T) vs Non-Tumor (NT), is shown by the third principal component (PC3) which explains 14.36% of the total variance in the dataset. Figure 2B and Figure S4 shows the grouping of variables into two separate clusters, i.e. T and NT. Differences across the different arms of the workflow has also affected PC3, which is illustrated by the grouping of TotalProtein (non-enriched) peptides in a single cluster in Figure 2B. Only patient 12 does not show any differences in T compared to NT according to Figure 2B. The PLS bi-plots demonstrate that there were no outliers in this dataset, as shown on the Hoteling T2-Range plot (Figure S3). PLS confirmed that the experiment was successful, and that there are significant differences between T and NT. Differences across the three different arms of the workflow exists, but TiO_2_ and IMAC have a nearly equal correlation. Together PC1, PC2 and PC3 explain 38.52% of the total variance in the dataset. The remaining variation in the dataset can be attributed to mixed effects of analytical and biological variability.

In addition to investigating raw isobaric tag intensities in T & NT specimens to identify outliers and groups, PLS-DA was also used to investigate the log_2_ T/NT ratios from all phospho-peptides (median from IMAC, TiO_2_, Non-enriched) in each case, as shown in Figure 2C. A subtle relationship between the grouping of cases and the time of recurrence appears to exit, however the number of biological repeats would need to be increased before coming to any final conclusions. That being said it is interesting to observe cases 14 and 9 grouped closely and both cases experienced very early recurrence at 2 and 5 months after surgery, respectively. Cases 10 and 8 also grouped together, but far away from all other cases. Case 10 showed recurrence at 31 months and case 8 had no sign of recurrence even 23 months post-surgery. Cases 4, 12, 1, 7, 5, and 13 grouped together and these showed recurrence between 10 to 21 months post-surgery. Interestingly case 6, which is yet to show recurrence, also grouped with the cases that showed recurrence at 10 to 21 months. Case 11 did not group with any other cases.

### Significantly regulated protein expression

**Figure 3:**
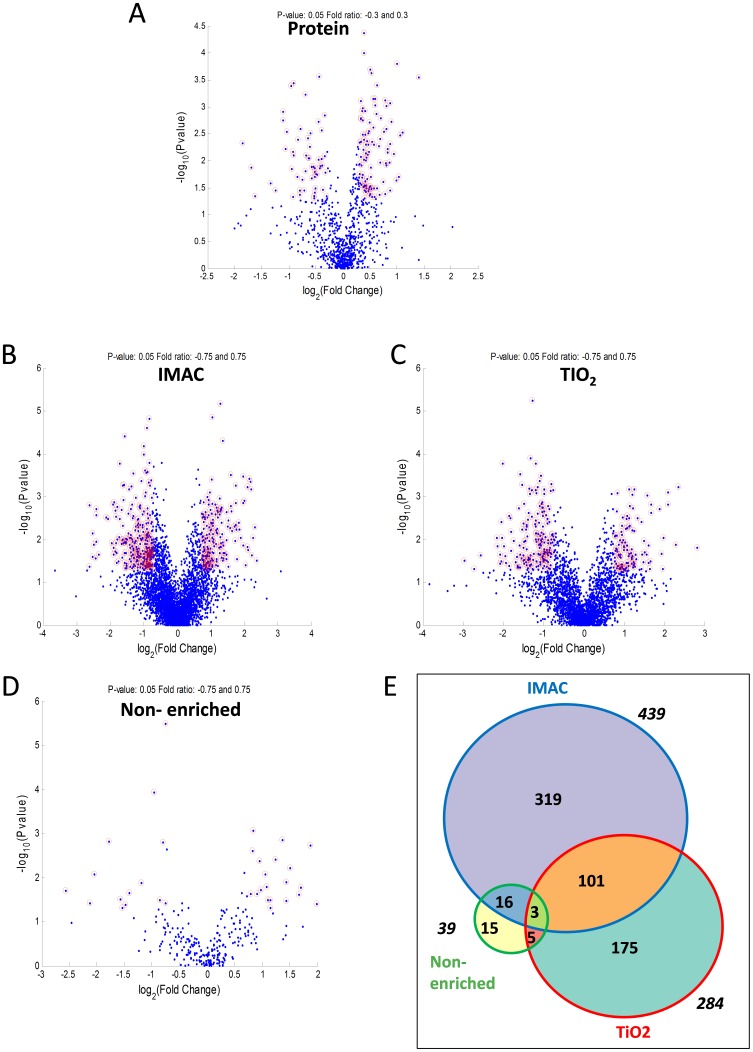
Number of significantly modulated proteins and phosphopeptides. Volcano plots showing −log_10_ P-values in relation to log_2_ T/NT ratios for; A: relative protein abundance (determined from median non-phosphopeptide log_2_ T/NT ratios), B: phosphopeptides measured in the IMAC, C: TiO_2_, D: and Non-enriched arm of the SysQuant workflow. Red circles point out significantly modulated proteins (log_2_ T/NT ratios ≥0.3 or ≤−0.3 and p-values ≤0.05) and phosphopeptides (log_2_ T/NT ratios ≥0.75 or ≤−0.75 and p-values ≤0.05). E: is a Venn diagram illustrating the distribution of the 635 phosphopeptides across the three arms of the workflow that were significantly modulated.

We determined the relative abundance of proteins in tumor compared to non-tumor tissue, using median log_2_ T/NT ratios of the non-phosphorylated peptides unique to each protein as surrogates to calculate the relative abundance of the respective proteins. A one sided t-test was used to calculate p-values and these were plotted against log_2_ T/NT ratios on a volcano plot to detect significant (Log_2_ T/NT≥0.3 or ≤−0.3 and p≤0.05) regulations over all cases (Figure 3A). In total there were 152 proteins significantly regulated based on Log_2_ T/NT≥0.3 or ≤−0.3 and p≤0.05 (File S6_Sheet ‘Pro_TvNT>or<0.3_p<0.05’). Table 2 displays the 12 most significantly upregulated proteins in tumor compared to non-tumor tissue, and also provides a description of any known function of each protein or association with cancer [13]–[31]. Overexpression of Mucin-1 is often associated with cancer and we also found Mucin-1 to be significantly up-regulated in pancreatic tumor tissue. Interestingly we found more significant up-regulated proteins than Mucin-1, some of which may prove to be more specific markers of pancreatic cancer, perhaps even new therapeutic targets e.g. Homeodomain-interacting protein kinase 1 (HIPK1). HIPK1, which was elevated in tumor compared to non-tumor in all cases (median log_2_ T/NT = 1.00; p = 1.59 E -04), is one of four HIPK serine/threonine kinases known to interact with and regulate the activity of numerous cellular proteins including several transcription factors and cofactors [32], [33], [34]. HIPKs have been implicated in the control of a range of cellular pathways to regulate various processes including the DNA damage response, tissue specification, and proliferation [34].

**Table 2:** The top 12 most significantly up-regulated proteins in tumor compared to non-tumor background tissue, on average over all 12 cases.

Uniprot-ID	Protein	p-values	log_2_ T/NT	Function	Role in cancer	References
P14618	Pyruvate kinase isozymes M1/M2	4.2E-05	0.383	Glycolytic enzyme that catalyzes the transfer of a phosphoryl group from phosphoenolpyruvate (PEP) to ADP, generating ATP	In addition to aerobic glycolysis, regulates gene transcription. Isoform M2 phosphorylates histone H3 at T11, which is related to expression of cyclin D1 and c-Myc, tumor cell proliferation, cell-cycle progression, and brain tumorigenesis.	Yang W, et al. Cell 2012. Christofk HR, et al. Nature 2008.
Q86Z02	Homeodomain-interacting protein kinase 1	1.6E-04	1.002	Belongs to the Ser/Thr family of protein kinases and HIPK subfamily. Phosphorylates p53, DAXX, and MYB. Prevents MAP3K5-JNK activation in the absence of TNF.	Known to be upregulated in many tumor cell lines. Involved in tumorigenesis and tumor growth by its oncogenic and anti-apoptotic function.	Kondo S, et al. Proc Natl Acad Sci USA 2003. Lee D, et al. EMPO Rep 2012.
Q14847	LIM and SH3 domain protein 1	2.0E-04	0.496	Plays an important role in the regulation of dynamic actin-based, cytoskeletal activities	Involved in proliferation, invasion and migration of cancer cells.	Zhao L, et al. Gut 2010. Grunewald TG, et al. Br J Cancer 2007.
P37802	Transgelin-2	2.3E-04	0.519	Contains a conserved actin-binding domain also known as the calponin homolog (CH) domain, suggesting a role in cytoskeletal organization.	Overexpressed in various cancers. Higher expression levels were associated with metastasis, advanced clinical stage, and poor survival. But its biological function remains unknown.	Zhang Y, et al. Cancer Sci 2010.
Q92538	Golgi-specific brefeldin A-resistance guanine nucleotide exchange factor 1	2.8E-04	1.397	Involved in mitosis. Phosphorylated by CDK1. Promotes the activation of ADP-ribosylation factor 5 (ARF5) through replacement of GDP with GTP.	Unknown.	Morohashi Y, et al. Biochem J 2010.
P21291	Cysteine and glycine-rich protein α1	4.0E-04	0.628	A cytoskeletal lin-11 isl-1 mec-3 (LIM)-domain protein. Involved in smooth muscle differentiation.	Down-regulated in hepatocellular carcinoma and colorectal cancer. But, its function is unknow.	Miyasaka KY, et al. Proc Natl Acad Sci U S A. 2007. Hirasawa Y, et al. Oncology 2006.
Q8WX93	Palladin	7.0E-04	0.588	Cytoskeletal protein that is required for organization of normal actin cytoskeleton. Roles in establishing cell morphology, motility, cell adhesion and cell-extracellular matrix interactions.	Overexpressed in breast cancer. Involved in cell migration. Plays a key role in the formation of podosomes, actin-rich structures that function in adhesion and matrix degradation.	Goicoechea SM, et al. Oncogene 2009.
Q14195-2	Isoform LCRMP-4 of Dihydropyrimidinase-related protein 3	7.0E-04	0.555	Necessary for signaling by class 3 semaphorins and subsequent remodeling of the cytoskeleton. Plays a role in axon guidance and cell migration	Unknown.	Weitzdoerfer R, et al. J Neural Transm Suppl. 2001.
Q9NR12	PDZ and LIM domain protein 7	7.4E-04	0.778	PDZ domain binds actin-binding proteins such as β-tropomyosin, while LIM domains interact with proteins involved in mitogenic or insulin signaling such as protein kinases. Involved in bone morphogenesis.	Promotes cell survival and chemoresistance by suppressing p53-mediated apoptosis. Elicited p53 degradation by inhibiting MDM2 self-ubiquitination and increasing its ubiquitin ligase activity toward p53 in cells.	Jung CR, et al. J Clin Invest 2010.
P26038	Moesin	7.6E-04	0.334	A membrane-cytoskeleton linking protein, belongs to the ERM (ezrin, radixin and moesin) family. Participates in various signaling pathways and play a crucial role in cell morphology, adhesion and motility.	Involved in actin filament remodelling and epithelial mesenchymal transition.	Haynes J, et al. Mol Biol Cell. 2011.
P15941	Mucin-1	8.6E-04	0.873	A transmembrane glycoprotein. The alpha subunit has cell adhesive properties. The beta subunit contains a C-terminal domain which is involved in cell signaling, through phosphorylation and protein-protein interactions.	An anti-adhesion molecule that inhibits cell–cell adhesion. Promoting motility and invasive properties by reducing interactions between integrins and the extracellular matrix. Involved in activation of Wnt and MAP signal pathways, and repression of the p53 gene.	Yonezawa, et al. Pathol Int 2011. Wei X, et al. Cancer Res 2007. Ren J, et al. J Biol Chem 2002.
Q05682	Caldesmon	9.4E-04	0.597	A cytoskeletal protein. Stabilizes actin filaments and involves in myosin-actin interaction. Plays an essential role during cellular mitosis and receptor capping.	Inhibitory effects on cell motility and migration. But phosphorylation at particular sites (i.e., S12) reduces the anti-migratory effect.	Schwappacher R, et al. J Cell Sci 2013. Mayanagi T, et al. J Biol Chem 2008.

Log_2_ T/NT ratios of the non-phosphorylated peptides from each protein were used as surrogates to calculate the relative abundance of the respective proteins. Log_2_ T/NT ratios of the non-phosphorylated peptides were averaged over three arms of the workflow (IMAC, TiO_2_, Non-enrich).

**Figure 4:**
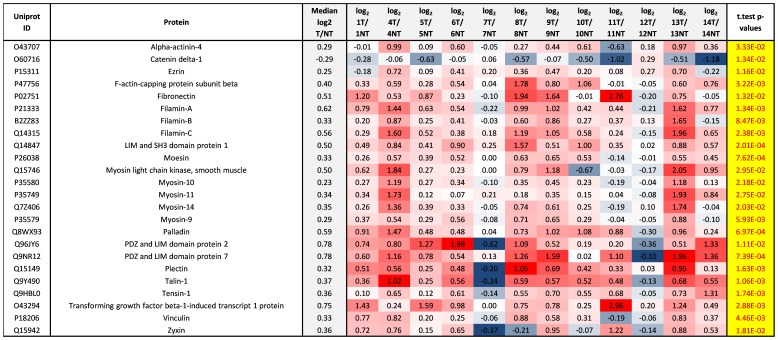
Significantly modulated lamellipodium and focal adhesion proteins. All proteins in this figure were shown to be associated with the GO terms ‘lamellipodium’ & ‘focal adhesion’ and also shown to be significantly (p≤0.05) up- or down- regulated in tumor compared to non-tumor tissue and quantifiable in each case (e.g. all proteins containing NA for any case were excluded from the table). Log_2_ T/NT ratios of the non-phosphorylated peptides from each protein were used as surrogates to calculate the relative abundance of the respective proteins. Log_2_ T/NT ratios of the non-phosphorylated peptides were averaged over three arms of the workflow (IMAC, TiO_2_, Non-enrich).

To better understand the biological processes and KEGG signaling pathways differing between tumor and non-tumor we selected the accession numbers of all significantly modulated proteins and uploaded these to the DAVID Bio-informatic resource. The Focal Adhesion KEGG signaling pathway was most significantly affected giving a Benjamini score of 1.0E-3. Significantly modulated Focal Adhesion proteins included; Talin-1, Filamin-A, Filamin-B, Filamin-C, Vinculin, Fibronectin, Zyxin, and Myosin light chain kinase, smooth muscle (Figure 4 & File S6_Sheet; FA & lamellipodium). Talin-2, Focal adhesion kinase 1 (FAK1), Protein phosphatase 1 regulatory subunit 12A were also focal adhesion proteins and significantly modulated but can only be seen in File S6, as these proteins were not quantifiable in some cases and Figure 4 only shows proteins quantifiable in all cases (e.g. no N/A). All of these focal adhesion proteins, except FAK1, were significantly up-regulated in tumor versus non-tumor suggesting increased size and/or frequency of focal adhesions in cells within tumor. Focal adhesions are known to play a role in migration of many cell types [35], [36], [37]. During migration the focal adhesions can anchor cells to the extracellular matrix following the formation of cell projections or protrusions; such as pseudopodium, filopodium, and lamellipodium [37]. The focal adhesion proteins Vinculin and Myosin Light Chain Kinase are also known to be involved in formation of lamellipodia and promote cell motility. On Figure 4 we list proteins known to be involved in formation of growth projections and focal adhesions, seen to be significantly modulated and measureable in all cases. The plasma membrane spanning extracellular matrix receptors (Integrins) are essential components of the focal adhesions however we did not observe statistically significant modulation of any integrin expression but did observe significant modulation of integrin phosphorylation, as discussed later. The assembly of focal adhesions also involves activation of Rho signaling as well as myosin-induced contractility [37]. Figure 4 also shows Myosin 9, 10, 11, and 14 were significantly elevated in tumor compared to non-tumor tissue.

Functional roles of four proteins in Figure 4 (LIM and SH3 domain protein 1, Moesin, Palladin, and PDZ and LIM domain protein 7) have already been discussed in table 2, but Alpha-actinin-4 (ACTN4) is an actin-binding protein with multiple roles in different cell types. In non-muscle cells, it is found along microfilament bundles and adherens-type junctions, where it is involved in binding actin to the membrane. It is believed to be involved in metastatic processes as Li Fu et al [38] demonstrated that overexpression of ACTN4 in combination with 67 LR is associated with Esophageal squamous cell carcinoma (ESCC) progression. They demonstrated that ACTN4 was differentially expressed in ESCC tissue compared to normal tissues and that expression levels of ACTN4 were progressively increased from stage I to III. Clinicopathological correlation using TMA revealed that overexpression of ACTN4 was significantly associated with advanced tumor stage (P = 2.6E-2) and lymph node metastasis (P = 4.9E-02) [38]. Plectin has also been proposed as a cancer biomarker, especially for pancreatic cancer [39]. Although normally a cytoplasmic protein, plectin is expressed on the cell membrane in pancreatic ductal adenocarcinoma (PDAC) and can therefore be used to target PDAC cells [39]. Our study confirms that both cancer biomarkers are significantly over expressed in tumor compared to non-tumor tissue in pancreatic cancer patients (median log_2_ T/NT = 0.29 and p-value = 3.33E-02 for ACTN4, and median log_2_ T/NT = 0.32 and p-value = 1.63E-03 for Plectin).

Catenin delta-1 is necessary to the formation of cell–cell adhesion (adherens junctions) through its interaction with the cytoplasmic tail of classical and type II cadherins. Catenin delta-1 also modulates the activities of the Rho family of GTPases (RhoA, Rac, and Cdc42), suggesting that along with other Src substrates, catenin delta-1 regulates actin dynamics. Thus, catenin delta-1 is a master regulator of adherens junction formation, and likely participates in regulating the balance between adhesive and motile cellular phenotypes [40]. Here we observe significantly decreased levels of the catenin delta-1 in tumor compared to non-tumor tissue (median log_2_ T/NT = −0.29 and p-value = 1.34E-02). When considering the important role catenin delta-1 plays in forming/maintaining adherens junctions between epithelial cells, and considering our observed decrease in expression and phosphorylation of this protein it suggests that these events may contribute to dissociation of epithelial cells, hence epithelial to mesenchymal transition in pancreatic cancer.

Of particular interest is discovering that Myosin light chain kinase (MLCK) is significantly overexpressed in tumor compared to non-tumor tissue (median log_2_ T/NT = 0.5 & p-value = 2.95E -02). MLCK is a Ca^2+^/calmodulin-dependent protein kinase that regulates a variety of cellular functions, such as, muscle contraction and cell migration, via phosphorylation of myosin light chain proteins. Since tumor cell migration is a key step in tumor spread, myosin light chain kinase (MLCK) may be regarded as a therapeutic target for preventing tumor spread. In fact, MLCK activation and expression have been found to be positively related with metastatic propensity. Moreover, MLCK inhibitors have been shown to diminish the invasiveness of various cancer cells [41]. Interestingly cases 14, 9, 4, and 13 have highest levels of MLCK and three out of the four of these cases also demonstrate very early recurrence (2 months, 5 months, 10 months, & the longest with 21 months recurrence, respectively). Perhaps these four cases would benefit from MLCK inhibitor therapy if patient stratification were based on high expression of the drug target in tumor versus non-tumor. Case 10 showed the lowest levels of MLCK in tumor compared non-tumor correlating with this case showing the longest time before recurrence of 31 months. MLCK also plays a role in p38 MAPK signaling a pathway demonstrating increased activity in several of the tumors in this study, as discussed later.

Observing increased Myosin expression in tumor tissue is also of particular interest as MYH9 (median log_2_ T/NT = 0.29 and p-value = 5.93E-03), MYH10 (median log_2_ T/NT = 0.23 and p-value = 2.18E-02), & MYH14 (median log_2_ T/NT = 0.35 and p-value = 2.03E-02) are all cellular myosins that are critical to cytokinesis, cell shape, and specialized functions such as secretion and capping. During cell spreading these three, play an important role in cytoskeleton reorganization, focal contacts formation (in the central part but not the margins of spreading cells), and MYH10 induces lamellipodial extension while this function is mechanically antagonized by MYH9, which is believed to cause lamellipodial retraction. MYH11 (median log_2_ T/NT = 0.34 and p-value = 2.75E-02) is a muscle cell myosin required for muscle contraction.

**Figure 5:**
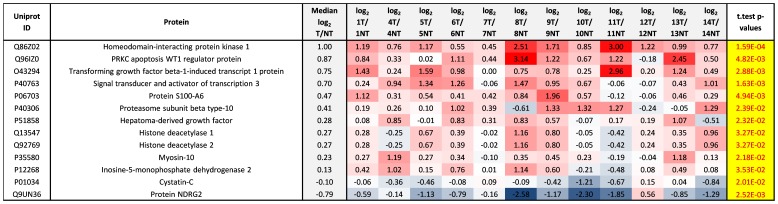
Significantly modulated proliferation proteins. All proteins in this figure were associated with the GO term ‘proliferation’ and also shown to be significantly (p≤0.05) up- or down- regulated in tumor compared to non-tumor tissue and quantifiable in each case (e.g. all proteins containing NA for any case were excluded from the table). Log_2_ T/NT ratios of the non-phosphorylated peptides from each protein were used as surrogates to calculate the relative abundance of the respective proteins. Log_2_ T/NT ratios of the non-phosphorylated peptides were averaged over three arms of the workflow (IMAC, TiO_2_, Non-enrich).

**Figure 6:**
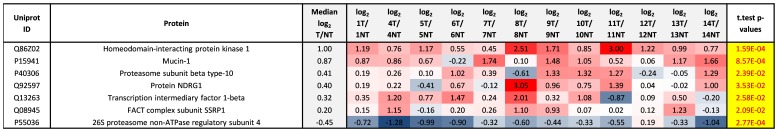
Significantly modulated DNA damage and repair proteins. All proteins in this figure were associated with the GO terms ‘DNA damage’ & ‘DNA repair’, and also shown to be significantly (p≤0.05) up- or down- regulated in tumor compared to non-tumor tissue and quantifiable in each case (e.g. all proteins containing NA for any case were excluded from the table). Log_2_ T/NT ratios of the non-phosphorylated peptides from each protein were used as surrogates to calculate the relative abundance of the respective proteins. Log_2_ T/NT ratios of the non-phosphorylated peptides were averaged over three arms of the workflow (IMAC, TiO_2_, Non-enrich).

In Figure 5 & 6, we select significantly modulated proteins quantifiable in all 12 cases and associated to the GO terms ‘proliferation’ and ‘DNA damage or DNA repair’, respectively. Again we see HIPK1 and Mucin-1 selected as proteins of interest however significant elevated tumor expression of several other proteins listed here such as STAT3, HDAC1&2, and Hepatoma derived growth factor also evoke interest especially as they are potentially effective therapeutic targets to anti-neoplastic agents.

**Figure 7:**
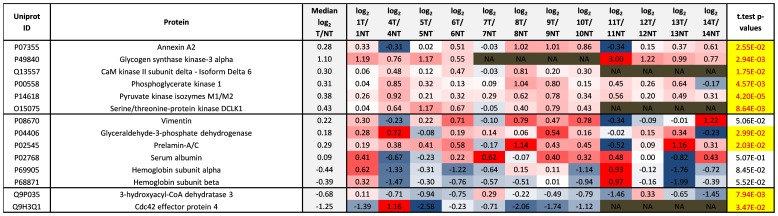
Mesenchymal (Vimentin), general cellularity and blood protein markers. Log_2_ T/NT ratios of the non-phosphorylated peptides from each protein were used as surrogates to calculate the relative abundance of the respective proteins. Log_2_ T/NT ratios of the non-phosphorylated peptides were averaged over three arms of the workflow (IMAC, TiO_2_, Non-enrich).

Sum scaling was used to normalise for any adverse effects on quantification from potential experimental or systematic bias. In Figure 7 we also display the relative abundance of blood proteins (Serum albumin and Hemoglobin A&B), the mesenchymal cell marker (Vimentin), and some cellularity markers (Glyceraldehyde 3-phosphate dehydrogenase & Prelamin A/C). We observe slightly more GAPDH and Prelamin A/C in the tumor tissue of most cases. Elevated GAPDH may be due to slightly higher cellularity or elevated glycolysis in tumor, yet elevated Prelamin A/C is more suggestive of elevated nuclear envelope and therefore cellularity. Six out of twelve of the cases display slightly elevated Vimentin while five out of twelve display slightly reduced levels of Vimentin. Vimentin was almost two fold higher in the tumor of case 14 suggesting high mesenchymal cell content in this tumor, perhaps relevant to case 14 early recurrence after only two months. A possible concern is the very different relative abundance of Haemoglobin and Serum albumin from case to case. These blood proteins are clearly very high in the non-tumor tissue of case 4 and case 13. Following protein extraction from tissue we performed protein assay to ensure equal protein amounts in each sample, however it seems much of the protein content from non-tumor tissue of case 4 and case 13 is from blood. The high content of blood proteins in the non-tumor tissue of case 4 and 13 may slightly skew the relative abundance ratios of other proteins perhaps explaining the particularly high levels of Myosins, Myosin light chain kinase, and Filamins in tumor tissue from case 4 and 13. For future investigations we will take steps to remove any residual blood.

### Significantly regulated phosphopeptides

**Figure 8:**
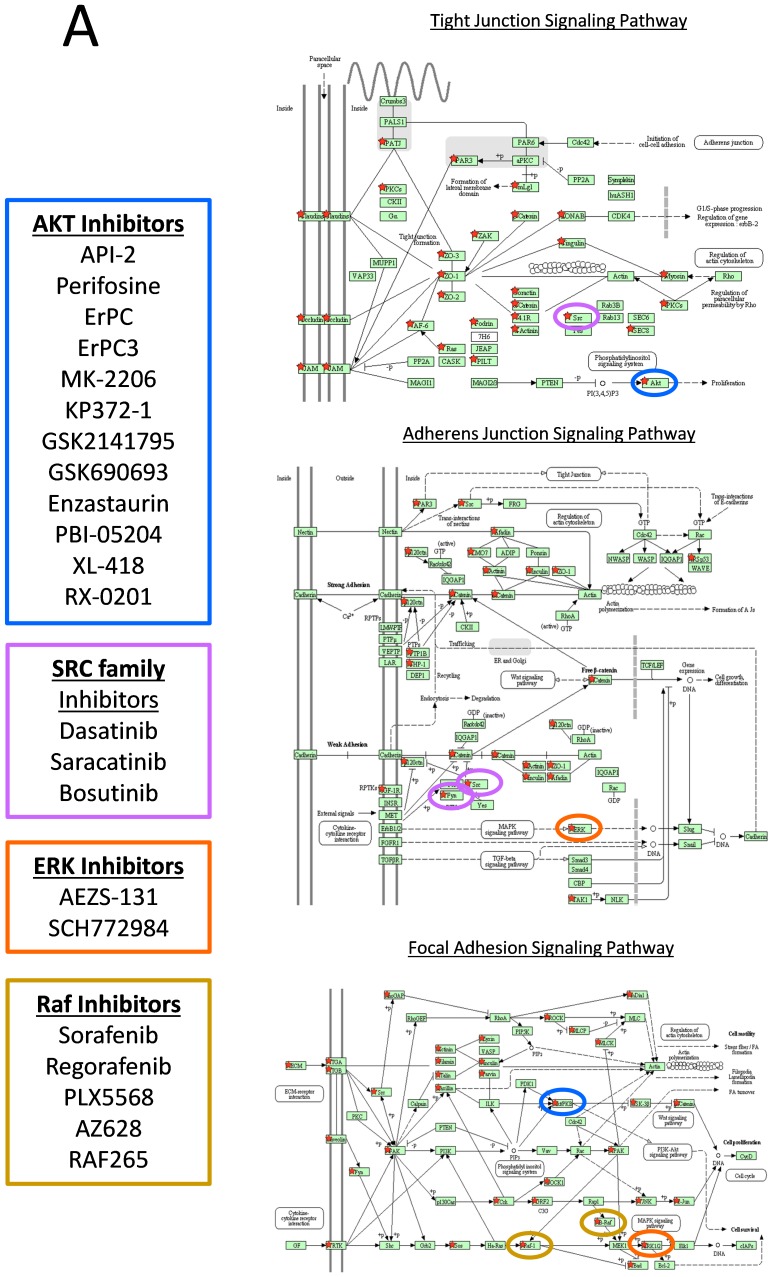
Signaling pathways highly modulated in pancreatic cancer. This schema summarizes all proteins identified as phosphorylated from the following KEGG signaling pathways; Tight Junction, Adherens Junction and Focal Adhesion. Red stars indicate those proteins identified as phosphorylated in any of 12 cases. Proteins highlighted by coloured circles are known drug targets.

**Figure 9:**
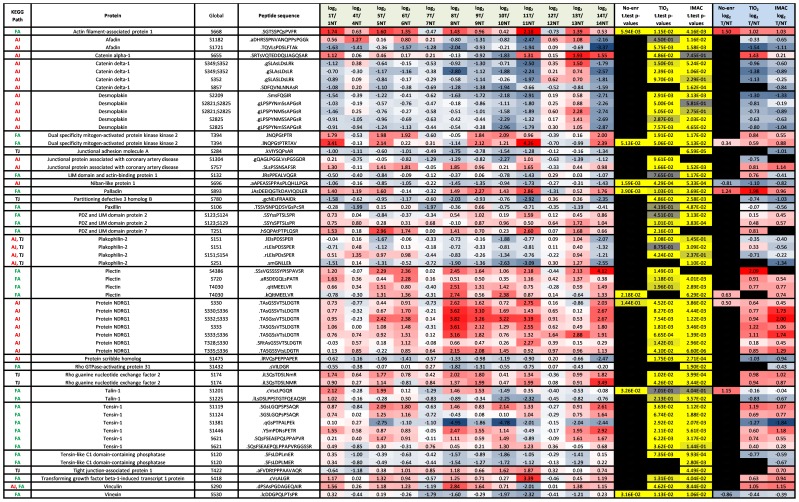
Significantly modulated phosphopeptides from key signaling proteins. All phosphopeptides here were significantly modulated in tumor compared to non-tumor tissue in at least one arm of the SysQuant workflow, quantifiable in all 12 cases, and from proteins shown to be associated with the Focal Adhesion (FA), Adherens Junction (AJ), and Tight Junction (TJ) KEGG signaling pathways. Here we display the KEGG pathway associated to the protein, the protein name, the global position of the phosphorylation site on the full length protein, the sequence of the quantified phosphopeptides where lower case s/t/y signifies the phosphorylated residues, the median log_2_ T/NT ratio over all three arms (non-enriched, TiO_2_ & IMAC) in each case, the t-test p-values calculated from all 12 cases for each arm of the workflow, and the median log_2_ T/NT ratio from all cases in either the non-enriched arm or TiO_2_ arm, or IMAC arm of the workflow.

P-values and log_2_ T/NT ratios for phosphopeptides were plotted on Volcano plots for IMAC, TiO_2_, and Non-enriched arms of the workflow, to detect significant (median log_2_ T/NT≥0.75 or ≤−0.75 and p≤0.05) regulations over all cases, as shown in Figure 3B–3D. Of the 5409 quantifiable phosphopeptides (File S4), 635 showed significant regulation (Figure 3B–3D) and these were from 408 unique proteins. The 408 protein accession numbers were uploaded to the DAVID bio-informatics resource which matched 14 of these proteins to the Tight Junction signaling pathway; the KEGG signaling pathway seen to be most significantly modulated in tumor relative to non-tumor (p = 2.50E-05). In addition to determining which phosphopeptides demonstrated significant differences in abundance between tumor and non-tumor tissue when averaged across all cases, we also wanted to determine which phosphopeptides were highly modulated on a case by case basis. Accession numbers of proteins which yielded phosphopeptides demonstrating log_2_ T/NT ratios of ≥1, or ≤−1 (More than 2 fold up-/down- regulated), were selected separately from each case. Accession numbers were then uploaded to the DAVID Bioinformatic resource which identified KEGG signaling pathways which matched with greatest significance for each case based on p-values and Benjamini scores (Table S7, in Tables S1). KEGG pathways in Table S7 in Tables S1 with Benjamini scores ≤0.05 were highlighted in Yellow. Based on p-values tight junction signaling pathway was determined to be modulated between tumor compared to non-tumor in all cases (12/12 cases), followed by adherens junction signaling (10/12 cases) and focal adhesion signaling (10/12). Figure 8 shows the tight junction, adherens junction and focal adhesion KEGG signaling pathways and the rectangles marked with red stars indicate those proteins we identified as phosphorylated across all 12 cases. We also highlight the known anti-cancer drug targets in these pathways as indicated in the figure legend. In Figure 9, we display the log_2_ T/NT ratios of all significantly modulated phosphopeptides from focal adhesion proteins, tight junction proteins and adherens junction proteins that were quantifiable in all twelve cases. We discuss some of these phosphorylation events and their potential roles in pancreatic cancer in more detail however it is beyond the scope of this study to discuss all. Our aim is to make all the data available to the reader, in the form of tables and supplemental files, but also select particular phosphorylation events we believe to be of greatest interest and discuss them here in more detail.

### Phosphorylation of proteins associated with cell-cell or cell-extracellular matrix (ECM) adhesions

On Figure 9 we show significantly elevated levels in tumor compared to non-tumor of the phosphopeptide containing the AFAP phosphorylation site S668 (median log_2_ T/NT = 1.50 and p-value = 5.94E-03 for Non-enrich, median log_2_ T/NT = 1.02 and p-value = 1.15E-02 for TiO_2_, median log_2_ T/NT = 1.03 and p-value = 4.16E-03 for IMAC). File S4 & S5 also show elevated levels of peptides containing the AFAP phosphorylation sites S664, S665, and S668, but no non-phosphorylated peptides were quantified therefore relative expression of AFAP could not be quantified. Actin filament-associated protein 1 (AFAP) is an actin cross-linking protein and has been shown to be significantly increased in prostate carcinomas relative to normal prostatic epithelium as well as benign prostatic hyperplasia [42]. Downregulation of AFAP has previously been shown to inhibit cell proliferation and tumorigenicity in pancreatic cancer cell lines and mouse models. Furthermore, down-modulation of AFAP can result in decreased cell-matrix adhesion and cell migration, defective focal adhesions, and reduced integrin beta-1 expression. Increased expression of AFAP is associated with progressive stages of prostate cancer and is critical for tumorigenic growth, in part by regulating focal adhesions in a PKC-dependent mechanism. Considering the important role AFAP plays in cancer and the significantly elevated levels of phosphorylated AFAP detected in pancreatic tumor tissue in this study, we believe AFAP and its phosphorylation is most likely an important player in pancreatic cancer particularly cell migration. It is not known which kinase causes phosphorylation at these particular sites however it is known that AFAP is a substrate of Src and PKC.

Catenin delta-1 was originally identified as a Src substrate, and here we observe significantly decreased tumor levels of the phosphopeptides containing the catenin delta-1 phosphorylation sites S349, S352, and S857 (Figure 9). As discussed earlier, quantification of non-phosphorylated catenin delta-1 peptides confirmed its expression was significantly decreased in tumor compared to non-tumor tissue, however the reduction in phosphorylation was more pronounced than the reduction in expression (File S4 & S5). We also quantified phosphopeptides containing the catenin delta-1 phosphorylation sites; S47, S252, S268, S269, S346, S861, and S864, however these were not significantly modulated (File S4). Decreased expression and phosphorylation of catenin delta-1 may play a role in epithelial to mesenchymal transition in pancreatic cancer.

Junctional adhesion molecule A (JAM-A) phosphorylation site S284 was decreased in tumor tissue compared to non-tumor tissue of all cases (median log_2_ T/NT = −1.01 & p-value = 6.59E-05 for IMAC). Phosphorylation of JAM-A at S284 is known to be a critical step in the formation and maturation of tight junctions [43]. Here we observe a significant decrease of JAM-A S284 phosphorylation in tumor tissue suggesting there is less tight junction formation between tumor cells an event that could favour epithelial to mesenchymal transition (EMT) of the cells and consequently metastatic spread. We did not quantify any non-phosphorylated JAM-A peptides however additional JAM-A phosphopeptides were quantified as shown on File S4 & S5.

Rho guanine nucleotide exchange factor 2 (GEF-H1) is a microtubule-associated guanine nucleotide exchange factor for Rho GTPases [44]. Here, in Figure 9, we show significant increase of the phosphopeptides containing GEF-H1 phosphorylation site S174 in tumor compared to non-tumor. No non-phosphorylated peptides from GEF-H1 were quantified so we were not able to quantify relative protein abundance (File S5), however we did quantify additional GEF-H1 phosphopeptides containing phosphorylation sites S152, S163, S177, S643, S645, and S932. The doubly phosphorylated peptide containing sites S174 and S177 were also significantly increased but only quantifiable in cases 1–10, not cases 11–14 (File S4). All GEF-H1 phosphopeptides except that containing phosphorylation site S643 were elevated on average in tumor compared to non-tumor. Guanine nucleotide exchange factors (GEFs) activate monomeric GTPases by stimulating the release of guanosine diphosphate (GDP) to allow binding of guanosine triphosphate (GTP) [44]. The aberrant activity of Ras homologous (Rho) family small GTPases (20 human members) has been implicated in cancer and other human diseases [44]. However, in contrast to the direct mutational activation of Ras found in cancer and developmental disorders, Rho GTPases are activated most commonly in disease by indirect mechanisms. One prevalent mechanism involves aberrant Rho activation via the deregulated expression and/or activity of Rho family guanine nucleotide exchange factors (RhoGEFs). Rho GTPases specifically regulate actin organization, cell motility (through formation of lamellipodia and filipodia), polarity, growth, survival and gene transcription [44]. Rho guanine nucleotide exchange factors (RhoGEFs), such as GEF-H1, accelerate the intrinsic exchange activity of Rho GTPases to stimulate formation of Rho-GTP [44]. File S4 shows some additional GEFs with significantly increased phosphorylation in tumor compared to non-tumor, including; Rho guanine nucleotide exchange factor 11 at S251 and Rho guanine nucleotide exchange factor 17 at S420 & S735. Inversely we observed significantly decreased phosphorylation of Rho guanine nucleotide exchange factor 12 at T703 & S1327 & Rho guanine nucleotide exchange factor 17 at S764. Here we also observe significant decrease in the phosphopeptide containing Rho GTPase-activating protein 31 phosphorylation site S1432. Modulation of phosphopeptides from several other Rho signal transduction proteins can be observed in File S4 by filtering for the GO term ‘Rho protein signal transduction’ in the column entitled ‘GeneOntologyGO’.

Integrin phosphopeptides were also observed to be significantly modulated however some of these phosphopeptides were not measureable in all cases (File S4 & S7). The doubly phosphorylated peptide containing the Integrin beta-4 phosphorylation sites S1483 and S1486, was elevated more than two fold in the tumor tissue compared to non-tumor tissue of case 1. This phosphopeptide was found to be significantly elevated in tumor tissue compared to non-tumor across all measured cases. Integrin beta-4 phosphorylation has been associated with the disassembly of cell anchoring junctions, such as hemidesmosomes at the trailing edge of migrating cells [45], [46]. Such phosphorylation events have been shown to be induced by Fyn (primarily at Tyrosine residues), PKC (primarily at Serine residues), and other kinases [45].

#### Catenin alpha-1

The singly phosphorylated peptide containing Catenin alpha-1 phosphorylation site S655 was elevated more than two fold in tumor tissue compared to non-tumor, in case 1 and in fact significantly elevated in tumor tissue on average across all cases (Figure 9). Phosphorylation at S641, S655, and S658, was elevated in tumor tissue of all but three cases (File S4), two of those three being stage IIA. Interestingly phosphorylation of catenin alpha-1 at S641 has been shown to lead to dissociation between catenin alpha-1 and catenin beta-1 (beta catenin), leading to increased transcriptional activation of beta-catenin and tumor cell invasion [47].

### Phosphorylation of protein kinases

**Figure 10:**
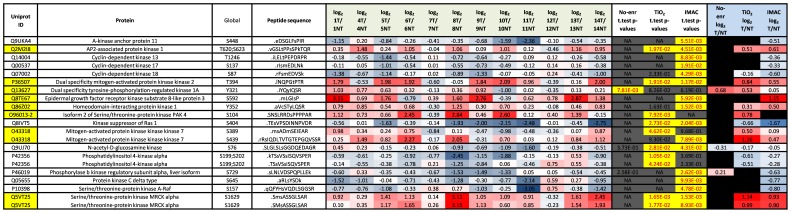
Significantly modulated phosphopeptides from key kinases. All phosphopeptides here were significantly modulated in tumor compared to non-tumor tissue in at least one arm of the SysQuant workflow, quantifiable in all 12 cases, and from proteins shown to contain the word ‘kinase’ in their protein name. Here we display the Uniprot accession number, the protein name, the global position of the phosphorylation site on the full length protein, the sequence of the quantified phosphopeptides where lower case s/t/y signifies the phosphorylated residues, the median log_2_ T/NT ratio over all three arms (non-enriched, TiO_2_ & IMAC) in each case, the t-test p-values calculated from all 12 cases for each arm of the workflow, and the median log_2_ T/NT ratio from all cases in either the non-enriched arm or TiO_2_ arm, or IMAC arm of the workflow.

In File S4, we filtered all proteins containing ‘kinase’ in their name and imported these to Figure 10 & File S7. The phosphopeptides in Figure 10 are quantifiable in all twelve cases and significantly modulated in tumor compared to non-tumor. Of particular interest was the observation that the phosphopeptides from Serine/Threonine-protein kinase MRCK alpha containing phosphorylation site S1629 were significantly elevated in tumor compared to non-tumor. In fact, only in case 12 did we not see an increase of this phosphopeptide in tumor tissue. In addition, File S7 (*Sheet; All Kinase phos*) shows that phosphopeptides containing MRCK alpha phosphorylation sites 1629, 1635, 1651, and 1654 were elevated in tumor relative to non-tumor, for most cases. MRCK alpha is an important downstream effector of the Rho GTPase, CDC42, and plays a critical role in the regulation of cytoskeleton reorganization, formation of cell protrusion, and promotes cell migration. The specific role of the phosphorylation event S1629 is not yet known. We were only able to determine relative expression of MRCK in Cases 7–10 (File S5). We can also see on Figure 10, that tumor tissue showed elevated phosphorylation of; AP2-associated protein kinase 1, Dual specificity mitogen-activated protein kinase kinase 2 (MEK 2), HIPK1, Serine/threonine-protein kinase PAK 4 (Isoform 2) and Mitogen-activated protein kinase kinase kinase 7 (TAK1). MEK2 is known to be downstream of RAS/RAF and upstream of ERK1/2, however there is no known function for the significantly up-regulated in tumor phosphorylation site T394. PAK4 is a serine/threonine protein kinase that plays a role in a variety of different signaling pathways including cytoskeleton regulation, cell migration, growth, proliferation and cell survival. Similarly to MRCK alpha, PAK4 is activated by GTPases CDC42 and RAC1 which results in a conformational change and a subsequent autophosphorylation on several serine and/or threonine residues, then activates the downstream target RHOA that plays a role in the regulation of assembly of focal adhesions and actin stress fibers.

Significantly elevated phosphorylation of TAK1 on S389 & S439 in tumor tissue relative to non-tumor is also highly interesting, as TAK1 is a serine/threonine kinase which acts as an essential component of the MAP kinase signal transduction pathway. TAK1 mediates signal transduction of TRAF6, various cytokines including interleukin-1 (IL-1), transforming growth factor-beta (TGFB), TGFB-related factors like BMP2 and BMP4, toll-like receptors (TLR), tumor necrosis factor receptor CD40 and B-cell receptor (BCR). It also induces activation of MKK/JNK signal transduction cascade and the p38 MAPK signal transduction cascade through the phosphorylation and activation of several MAP kinase kinases. Considering the role TAK1 plays in activating signaling cascades, we believe phosphorylation of TAK1 at S389 & S439 may be of importance in pancreatic cancer.

### Phosphorylation of proteins associated with GO terms ‘DNA damage’ & ‘DNA repair’

**Figure 11:**
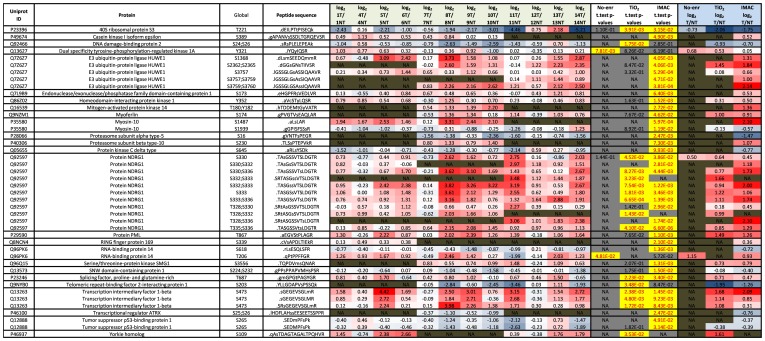
Significantly modulated phosphopeptides from DNA damage or repair proteins. All phosphopeptides here were significantly modulated in tumor compared to non-tumor tissue in at least one arm of the SysQuant workflow, and from proteins associated to the GO terms ‘DNA damage’ or ‘DNA repair’. Here we display the Uniprot accession number, the protein name, the global position of the phosphorylation site on the full length protein, the sequence of the quantified phosphopeptides where lower case s/t/y signifies the phosphorylated residues, the median log_2_ T/NT ratio over all three arms (non-enriched, TiO_2_ & IMAC) in each case, the t-test p-values calculated from all 12 cases for each arm of the workflow, and the median log_2_ T/NT ratio from all cases in either the non-enriched arm or TiO_2_ arm, or IMAC arm of the workflow.

Ten out of the twelve patients experienced tumor recurrence at various time points after surgery and gemcitabine chemotherapy. In fact 95% of patients with pancreatic cancer are expected to show recurrence within 5 years of surgery and chemotherapy, therefore even though cases 6 and 8 are yet to experience recurrence we would expect this to occur eventually. Gemcitabine is a nucleoside analogue used as chemotherapy. As with fluorouracil and other analogues of pyrimidines, the drug replaces one of the building blocks of nucleic acids, in this case cytidine, during DNA replication. The process arrests tumor growth, as new nucleosides cannot be attached to the “faulty” nucleoside, resulting in apoptosis (cellular “suicide”). Clearly however, the inevitable problem of recurrence means the DNA damage induced by gemcitabine is not always catastrophic enough to induce apoptosis in all pancreatic cancer cells. Usually following DNA damage the cells sense this due to DNA damage sensing proteins which activate DNA repair mechanism and if the damage is too great then they trigger apoptosis. Sometimes however the DNA repair mechanisms can rescue the cancer cells from apoptosis therefore leading to chemotherapy resistance and ultimately recurrence and death. This resistance can be seen in glioblastoma patients receiving DNA damaging chemotherapies who overexpress the DNA repair enzyme (O^6^-methylguanine-DNA-methyltransferase (MGMT)); whereas patients with hyper-methylation of the MGMT gene respond much better to these alkylating chemotherapies. Epigenetic silencing of the MGMT gene by methylation of the CpG islands of the promoter region has been shown to correlate with loss of gene transcription and protein expression. Loss of expression of the MGMT protein results in decreased DNA repair and retention of alkyl groups, thereby allowing alkylating agents such as carmustine (BCNU), lomustine (CCNU), and temozolomide to have greater efficacy in patients whose tumors exhibit hypermethylation of the MGMT promoter and reducing the MGMT protein concentration. [48]. This relationship between decreased expression of a DNA repair protein (MGMT) and better response to alkylating chemotherapies lead us to investigate the expression and phosphorylation status of DNA damage sensing proteins and DNA repair proteins in our dataset. In File S4, we filtered all proteins associated with the GO terms ‘DNA damage’ and/or ‘DNA repair’, then exported all of those significantly modulated phosphopeptides to Figure 11. Interestingly we identify two additional kinases (Mitogen-activated protein kinase 14 & Serine/Threonine-protein kinase SMG1) that were not shown in Figure 10, as Figure 10 only contained phosphopeptides that were quantifiable in all 12 cases. Mitogen-activated protein kinase 14 (MAP kinase p38 alpha) & Serine/Threonine-protein kinase SMG1 (SMG-1) both contained significantly elevated phosphopeptides in tumor compared to non-tumor however these were only quantified in cases 7–10 and cases 7–14, respectively. MAP kinase p38 alpha is activated by cell stresses such as DNA damage and heat shock, as well as pro-inflammatory stimuli such as interleukin-1. Activation occurs through dual phosphorylation of Thr-180 and Tyr-182 by either of two dual specificity kinases, MEK3 or MEK6, and potentially also MEK4. MAP kinase p38 alpha phosphorylated on both Thr-180 and Tyr-182 is 10–20-fold more active than MAP kinase p38 alpha phosphorylated only on Thr-180, whereas MAP kinase p38 alpha phosphorylated on Tyr-182 alone is inactive. Figure 11 demonstrates that MAP kinase p38 alpha must be greater than two fold more active in tumor tissue of cases 8, 9 & 10, compared to their respective non-tumor tissue.

### Phosphorylation events that indicate activation status of drug targets

**Figure 12:**
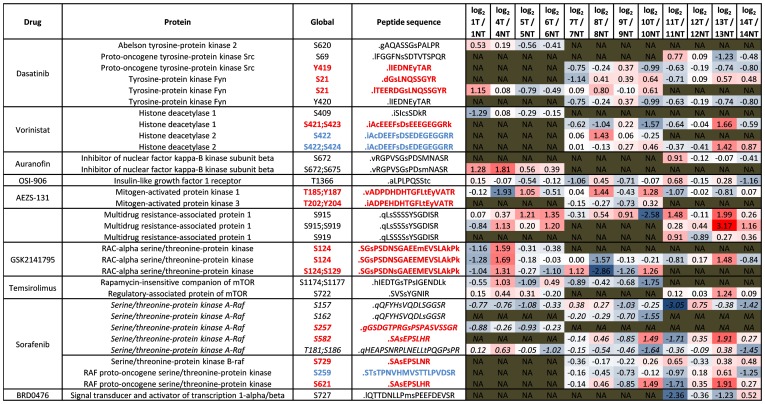
Phosphorylation indicates activity of drug targets. Here are examples of phosphopeptides that contain activator and inhibitor phosphorylation sites on proteins known to be anti-cancer drug targets. Here we display the inhibitory drug, the protein name, the global position of the phosphorylation site on the phosphoprotein, and the sequence of the phosphopeptide. The phosphorylated s/t/y residue in each peptide sequence is in lower case. The log_2_ T/NT ratios displayed in each case were median values calculated from all three arms of the workflow. Phosphopeptides in red contain activator phosphorylation sites, while phosphopeptides in blue contain inhibitor phosphorylation sites. Phosphopeptides in black contain phosphorylation sites with no known function.

To ascertain relative activation status of known drug targets in tumor compared to non-tumor tissue in each case, we used relative abundance of phosphopeptides containing phosphorylation sites known to either induce enzyme activation or inhibition of such drug targets. Figure 12 short lists all such phosphopeptides.

#### Fyn

The relative abundance of the peptide containing phospho-S21 of the Tyrosine-protein kinase Fyn is elevated more than two fold in tumor tissue compared to non-tumor tissue of case 1 (Figure 12). Phosphorylation of Fyn at serine 21 is reported to activate Fyn kinase [49]. This suggests therefore, that Fyn is more active in the tumor tissue compared to non-tumor tissue of case 1. Interestingly, phospho-serine 21 of Fyn is detected in all 12 cases, but it is only in cases 1, 8, 10, 13, and 14, that we observe elevated levels in tumor compared to non-tumor. Inversely, the tumor tissue of case 7 shows over a two-fold decrease of this phosphopeptide compared to non-tumor tissue. Fyn is a target of the approved kinase inhibitor Dasatinib therefore measurement of the peptide containing phospho-S21 using our workflow may be an attractive predictive marker for this drug.

#### MAPK1 (ERK2)

The relative abundance of the peptide containing phospho-T185 and phospho-Y187 of MAPK1 is elevated more than two fold in tumor tissue compared to non-tumor tissue of cases 5, 8, and 10 (Figure 12 and Table S8, in Tables S1). Phosphorylation of MAPK1 at T185 and/or Y187 is reported to activate MAPK1 [50]. This suggests therefore, that MAPK1 is more active in the tumor tissue compared to non-tumor tissue of cases 5, 8, and 10. Inversely, the tumor tissue of cases 4 and 11 shows more than two fold reduction of this phospho-T185 and phospho-Y187 containing phosphopeptide, compared to non-tumor tissue. MAPK1 is an anti-cancer drug target (AEZS-131 and SCH772984) and is also down-stream of many other anti-cancer drug targets (Anti-HER TKIs, Anti-MEK KIs), therefore measurement of the peptide containing phospho-T185 and phospho-Y187 using our workflow may be a predictive marker for these targeted anti-cancer therapies. We have also measured the singly phosphorylated peptides containing phospho-T185 or phospho-Y187, as well as the MAPK2 (ERK1) doubly and singly phosphorylated peptides containing phospho-T202 and phospho-Y204. SysQuant enables us to determine whether MAPK2 is phosphorylated on T202 and/or Y204 and/or MAPK1 is phosphorylated on T185, and/or Y187, yielding critical signaling pathway activation status information, unattainable by western blotting and other antibody based assays.

#### AKT1

The relative abundance of the singly phosphorylated peptides containing phospho-S124 and the doubly phosphorylated peptide containing phospho-S124 and phospho-S129 of AKT1 are elevated more than two fold in tumor tissue compared to non-tumor tissue of cases 4, 7, 10, and 13 (Figure 12 and Table S8, in Tables S1). Phosphorylation of AKT1 at S124 and/or S129 is reported to activate AKT1 [51], [52]. This suggests that AKT1 is more active in the tumor tissue compared to non-tumor tissue of cases 4, 7, 10, and 13, therefore anti-AKT kinase inhibitors may be effective in these patients. Interestingly Case 10 also demonstrated elevated MAPK1 activity suggesting this patient may be a candidate for dual AKT1 & MAPK1 inhibitor treatment, as such combination strategies have proven efficacy in pancreatic cancer cell lines and xenograft models [12]. Inversely, the relative lower abundance of phosphopeptides containing these activator phosphorylation sites suggests AKT1 is less active in the tumor tissue compared to non-tumor tissue of cases 1, 6, 8, 9, 11, and 14.

#### RAF1 & BRAF

Both are targets of Sorafenib the approved targeted therapy for advanced renal cell carcinoma and advanced hepatocellular carcinoma (HCC). The phosphopeptides containing the activator phosphorylation site S621 on RAF1 was elevated more than two fold in tumor compared to non-tumor of cases 10 and 13, suggesting elevated RAF1 activity in these cases. In tumor of case 14 there appears to be subtle increase in the activator phosphorylation sites S621 on RAF1 and S729 on BRAF, yet strong decrease in the inhibitor phosphorylation site S259 on RAF1, suggesting RAF1 and BRAF are more active in the tumor tissue of case 14. In future studies we plan to determine whether RAF1 and BRAF phosphorylation status serve as predictive markers to Sorafenib therapy in patients with advanced HCC. We also show ARAF phosphorylation on Figure 12, despite ARAF not being a known drug target of Sorafenib.

#### GSK3α

The peptide containing the Glycogen synthase kinase-3 alpha phosphorylation site Y279 increased more than two fold in the tumor tissue compared to non-tumor tissue of cases 1, 6, 13, and 14 (Table S8, in Tables S1, and File S4). Phosphorylation of Y279 causes activation of GSK3a which then induces cell survival, and reduces glycogen production [53]. GSK3a expression was measured in 8 out of 12 cases and shown to be significantly over expressed on average in tumor.

The relative abundance of phosphopeptides containing activator or inhibitor phosphorylation sites can help determine the relative activation status of; GSK3α and β, Histone deacetylase 1 and 2, RAF proto-oncogene serine/threonine-protein kinase, Serine/threonine-protein kinase A-Raf, Dual specificity mitogen-activated protein kinase kinase 6, Mitogen-activated protein kinase 14 (p38 MAPK), and over 20 others (File S4).

Several limitations need to be considered in this study. First, phosphorylation status of tissue might have been modified during sample collection. For example, although tissue samples were snap frozen 30 minutes after resection, one cannot exclude the possibility that ischaemia might have affected the phosphorylation status, however our quality control steps such as PLS-DA suggests our data has not been adversely affected by such issues. Second, as whole pieces of tissue were analysed, we cannot determine which phosphopeptides derives from cancer or stromal cells. However, considering that not only cancer cells but also stromal components are potential drug targets (i.e., inhibitors of angiogenesis), whole tissue samples may prove more helpful in understanding activated signaling pathways in cancer and to identify potential drug targets than micro-dissected cancer cells. Third, a common theme to current data dependent mass spectrometry is its ability to define the composition of complex proteomes/phosphorylomes and the functions of these complexes however “undersampling” still exists, that is, only a subset of the peptides will be identified if the complexity of the peptide sample exceeds the analytical capacity of the mass spectrometer e.g., when more peptides elute from the HPLC column per unit time than can be analyzed, or low-abundance peptides are below the instrument detection limit [54]. The undersampling issue (e.g presence of non-availables (N/A)) may explain why we can identify peptides in some but not all of the three TMT 8-plex analysed in this study. Future instrumentation and fractionation techniques will lead to complete proteome analysis eradicating undersampling [9].

In summary, we show examples here demonstrating how our LC-MS/MS proteomic workflow (SysQuant) can simultaneously measure the expression and phosphorylation of 1000's of proteins in tumor tissue relative to non-tumor tissue, and show how such measurements can be used to better understand the molecular events leading to cancer, and therefore the most suitable inhibitory agents, to treat a patient on a case by case basis. Within the field of LC-MS/MS based proteomics there are many groups developing very high quality analytical workflows to measure global protein expression and phosphorylation, however most studies from these groups are focused purely on either improving the sample preparation, or improving the LC-MS/MS analysis, or improving the data analysis software tools (computational mass spectrometry and/or bioinformatics), but rarely are these advancements and improvements grouped into a seamless workflow and applied to unravel the molecular events inducing cancer in a clinical setting. We believe this study builds on and further improves the technological advances in the field of LC-MS/MS proteomics and demonstrates how we can translate these into clinical oncology applications. This study demonstrates that LC-MS/MS proteomic workflows have the potential to become clinical tests and may improve clinical outcome for pancreatic cancer patients, as well as other cancers. In future we aim to improve SysQuant by reducing the turnaround time to two weeks (from receiving tissue specimen to interpreting data), reducing required specimen size from resected tissue to core needle biopsy, and also reducing economic cost. These are some examples of essential improvements to the current workflow for future applicability as a routine clinical test.

Throughout the manuscript we selected proteins and phosphopeptides significantly modulated then determined whether these proteins are known to contribute to migration, invasion, proliferation and/or DNA damage/DNA repair. Of particular interest was the observation of significantly increased expression of HIPK1 & MLCK, as well as observing significant increase in phosphorylation of the Serine/threonine-protein kinase MRCK alpha; as all three proteins may serve as effective new therapeutic targets. Despite these significant increases in tumor on average across all cases, we feel it is also important to highlight the interpatient variability in the expression and phosphorylation of these and other proteins e.g. cases 11 & 8 demonstrate substantially higher levels of HIPK1 in tumor (log_2_ T/NT of 3.00 & 2.51, respectively), while cases 7 & 6 do not show such high expression (log_2_ T/NT of 0.45 & 0.55, respectively); cases 13 & 4 demonstrate substantially higher levels of MLCK in tumor (log_2_ T/NT of 2.05 & 1.84, respectively), while cases 10 & 12 show reduced expression in tumor (log_2_ T/NT of −0.67 & −0.17, respectively); and finally cases 8 & 14 demonstrate substantially higher levels of phosphorylation of MRCKα in tumor (log_2_ T/NT>3.00 & >2.00, respectively), while case 12 shows reduced phosphorylation in tumor. This interpatient variability again highlights the need for suitable analytical capabilities, such as SysQuant, to determine the molecular events likely contributing to cancer from patient to patient, to then design more appropriate and bespoke treatment strategies for each case. Due to the volume of data, we have not been able to investigate the importance of every interesting molecular event observed here, and therefore invite experts from the oncology community focussed on specific areas of research to download our supplemental files (especially Files S4, S5, S6, and S7) and identify molecular events they feel also contribute to the cancer phenotype and therefore hopefully develop more effective therapeutics specific to inhibiting cancer. Our data gives a unique insight into the expression levels and phosphorylation status of thousands of proteins in clinical tumor tissue relative to the non-tumor background tissue. This data can be used to help validate theories and proposed mechanisms originating from functional investigations in model systems or the pre-clinical setting.

## Supporting Information

Figure S1 A: This MA-plot shows the log ratios vs. the log intensities over the complete non-normalized data set. B: This MA-plot shows the same as Figure S1A, but the data was normalized by sum-scaling and therefore better zero-centred.
(PPTX)

Figure S2 **Number of unique phosphopeptides and non-phosphopeptides identified in each raw file, from each SCX fraction, in each arm of the workflow (non-enrich, TiO2, and IMAC), from each TMT 8-plex sample (TMT 8-plex-1 shown in S2A, TMT 8-plex-2 shown in S2B, & TMT 8-plex-3 shown in S2C).** Most of the non-phosphorylated peptides eluted in fractions 7 to 11, while the phosphorylated peptides started to elute earlier but were more evenly distributed throughout the chromatography run time, except for a clear spike in the elution of phosphopeptides in fraction 6 from TMT 8-plex-1,2&3 (IMAC), fraction 6 from TMT 8-plex-2&3 (TiO2), and fraction 5&6 from TMT 8-plex-1 (TiO2).
(PPTX)

Figure S3 A: Hoteling T-Range plot shows no outlier's at the T2 plot. B: PC1 and PC2 Score plot of the first two principal components describing 13.6% (PC1) and 10.6% (PC2) of the total variance in the data. The circle depicts the T2 hotelling space based on 95% confidence. C: PLS Loading-plot PC1 and PC2.
(PPTX)

Figure S4 A: PLS Loading plot PC2 and PC3. B: PC2 and PC3 Score plot of the next principal components describing 10.6% (PC2) and 14.4% (PC3) of the total variance in the data. C: Here we zoom in on the total protein (non-enriched) cluster in Figure S4B. D: The same Score plot like Figure S4B, only the enrichment description in the label was deleted. E: Here we zoom in on the total protein (non-enriched) cluster in Figure S4D.
(PPTX)

File S1 **This is a zip excel file containing a list of all peptides (phosphorylated and non-phosphorylated) identified from all eight specimens in the TMT 8-plex-1 sample.** The file displays detailed information including; Sequest Xcorr, Mascot ions scores, ΔM [ppm], Percolator q-values, pRS-probabilities, raw quantification values, and other important information.
(7Z) Click here for additional data file.

File S2 **This is a zip excel file containing a list of all peptides (phosphorylated and non-phosphorylated) identified from all eight specimens in the TMT 8-plex-2 sample.** The file displays detailed information including; Sequest Xcorr, Mascot ions scores, ΔM [ppm], Percolator q-values, pRS-probabilities, raw quantification values, and other important information.
(7Z) Click here for additional data file.

File S3 **This is a zip excel file containing a list of all peptides (phosphorylated and non-phosphorylated) identified from all eight specimens in the TMT 8-plex-1 sample.** The file displays detailed information including; Sequest Xcorr, Mascot ions scores, ΔM [ppm], Percolator q-values, pRS-probabilities, raw quantification values, and other important information.
(7Z) Click here for additional data file.

File S4 **In this excel file we display all measurable phosphopeptides (5409) in all cases (12) from all arms of the workflow (non-enrich, IMAC, TiO2).** We display the Uniprot accession number, full protein name, phosphorylated residue number on full length protein, identified peptide sequence, confidence score of phosphorylated residue (pRS probability), t-test p-values for each arm of the workflow, and log_2_ T/NT ratios for each phosphopeptide on average across all cases, and in each individual case. We also display biological/functional relevant information if known to each phosphosite (from PhosphoPhositePlus), and to each protein (from GO and DrugBank databases).
(XLSX)

File S5 **In this excel file we display all measurable phosphopeptides but also display the relative protein abundance.** Relative protein abundance was determined by measuring the relative abundance of all non-phosphorylated unique to a specific protein (non-shared/non-homologous peptides). Here we also normalise phosphopeptide levels to relative protein abundance.
(XLSX)

File S6 **All proteins listed in this excel file were shown to be significantly up- or down-regulated in tumor compared to non-tumor tissue.** In the first sheet, proteins were deemed significantly modulated if they displayed p-values≤0.05. In the second sheet of the file, proteins were deemed significantly modulated if they displayed log_2_ T/NT≤−0.3 or ≥0.3 & p-values≤0.05. In the third sheet of the file proteins were deemed significantly modulated if they were measureable in tumor and non-tumor of all twelve cases (e.g. no non-available) and displayed log_2_ T/NT≤−0.3 or ≥0.3 & p-values≤0.05. The other sheets in this excel file contained significantly modulated proteins associated to the GO terms, DNA damage and repair, proliferation, focal adhesions and lamellipodia, and others.
(XLSX)

File S7 **Here, in the first sheet of this excel file, we display all significantly modulated phosphopeptides from proteins associated with Tight Junctions, Adherens Junctions, and Focal Adhesions.** In the second sheet we display Integrin phosphopeptides. In the third sheet we display all phosphophopeptides from Kinases. In the fifth sheet we filter significantly modulated phosphopeptides (p≤0.05) from kinases which were measureable in all specimens. In the sixth sheet we display all phosphopeptides from DNA repair proteins and in the seventh sheet filter all those phosphopeptides from DNA repair proteins which were significantly modulated (p≤0.05). In the eighth sheet we display all those phosphopeptides from proteins associated with the GO term ‘migration’.
(XLSX)

Methods S1 **This document contains supplemental methods.**
(DOCX)

Tables S1 **This document contains Tables S1–S8.** Table S1, Fourteen cases of pancreatic head ductal adenocarcinoma were selected from Institute of Liver Studies BioBank. Specimens from cases 2 and 3 yielded low protein amounts during protein extraction therefore were omitted from the study. Table S2, Information on tumor stage and recurrence are shown here. Yellow cases showed recurrence between 2 & 31 months after tumor removal. The difference between stage IIA and IIB is only the presence or absence of lymph node metastasis. Table S3, Additional non-confidential clinical information about patient and tumor. Table S4, Protein amounts from each sample per TMT 8-plex, used for the SysQuant workflow in this study. Table S5, Peptides from each specimen are labelled with different tandem mass tags (TMT). All peptides from case 1 tumor, case 10 non-tumor, and case 11 tumor were labelled with the 126 Da tandem mass tag (TMT) while peptides from case 1 non-tumor, case 10 tumor and case 11 non-tumor were labelled with the lighter 127 Da tandem mass tag (TMT), and so on as shown below. The lighter 127 (127e) and heavier 127 only differ in mass by 6 milli-Daltons, as do 129e and 129 reporter ions. Table S6, Nine aliquots of TMT labelled peptides were separated by SCX-HPLC. Table S7, Accession numbers of proteins which yielded phosphopeptides demonstrating log_2_ T/NT ratios of ≥1, or ≤−1 (More than 2 fold up/down- regulated), were selected separately from each case. Accession numbers were then uploaded to the DAVID Bioinformatic resource (separately for each case) which identified KEGG signaling pathways matched with greatest significance based on p-values and Benjamini scores. KEGG pathways with Benjamini scores ≤0.05 were highlighted in Yellow. Table S8, Case by case – Here we selected all phosphopeptides displaying log_2_ T/NT ratios ≥1 or ≤−1, that also contain phosphorylation sites that are known to either induce activation or inhibition of the phosphorylated enzyme (based on PhosphoSitePlus database). This was done for each case, on a case by case basis. T/NT is the average log_2_ ratio of phosphopeptide in tumor versus background tissue observed across all three arms of the workflow (IMAC, TiO2, Non-enrich). We also indicate whether the enzyme is a drug target based on the Drug Bank database.
(DOCX)

## References

[1] Smart JE, Oppermann H, Czernilofsky AP, Purchio AF, Erikson RL, et al (1981) Characterization of sites for tyrosine phosphorylation in the transforming protein of Rous sarcoma virus (pp60v-src) and its normal cellular homologue (pp60c-src). Proc. Natl. Acad. Sci USA 78: 6013–6017. 10.1073/pnas.78.10.6013 PMC348967 6273838

[2] Langer T, Vogtherr M, Elshorst B, Betz M, Schieborr U, et al (2004) NMR backbone assignment of a protein kinase catalytic domain by a combination of several approaches: application to the catalytic subunit of cAMP-dependent protein kinase. Chembiochem 5: 1508–1516. 15481030 10.1002/cbic.200400129

[3] Chen PL, Scully P, Shew JY, Wang JY, Lee WH (1989) Phosphorylation of the retinoblastoma gene product is modulated during the cell cycle and cellular differentiation. Cell 58: 1193–1198. 2673546 10.1016/0092-8674(89)90517-5

[4] Bononi A, Agnoletto C, De Marchi E, Marchi S, Patergnani S, et al (2011) Protein kinases and phosphatases in the control of cell fate. Enzyme Res 2011: 329098. 21904669 10.4061/2011/329098 PMC3166778

[5] Bond-Smith G, Banga N, Hammond TM, Imber CJ (2012) Pancreatic adenocarcinoma. BMJ 344: e2476. 22592847 10.1136/bmj.e2476

[6] Michl P, Gress TM (2013) Current concepts and novel targets in advanced pancreatic cancer. Gut 62: 317–326. 23112132 10.1136/gutjnl-2012-303588

[7] Llovet JM, Ricci S, Mazzaferro V, Hilgard P, Gane E, et al (2008) SHARP Investigators Study Group. Sorafenib in advanced hepatocellular carcinoma. N Engl J Med 359: 378–390. 18650514 10.1056/NEJMoa0708857

[8] Engholm-Keller K, Larsen MR (2013) Technologies and challenges in large-scale phosphoproteomics. Proteomics 13: 910–931. 23404676 10.1002/pmic.201200484

[9] Mann M, Kulak NA, Nagaraj N, Cox J (2013) The coming age of complete, accurate, and ubiquitous proteomes. Mol Cell 49: 583–590. 23438854 10.1016/j.molcel.2013.01.029

[10] McAlister GC, Huttlin EL, Haas W, Ting L, Jedrychowski MP, et al (2012) Increasing the multiplexing capacity of TMTs using reporter ion isotopologues with isobaric masses. Anal Chem 84: 7469–7478. 22880955 10.1021/ac301572t PMC3715028

[11] Werner T, Becher I, Sweetman G, Doce C, Savitski MM, et al (2012) High-resolution enabled TMT 8-plexing. Anal Chem 84: 7188–7194. 22881393 10.1021/ac301553x

[12] di Magliano MP, Logsdon CD (2013) Roles for KRAS in Pancreatic Tumor Development and Progression. Gastroenterology 144: 1220–1229. 23622131 10.1053/j.gastro.2013.01.071 PMC3902845

[13] Yang W, Xia Y, Hawke D, Li X, Liang J, et al (2012) PKM2 phosphorylates histone H3 and promotes gene transcription and tumorigenesis. Cell 150: 685–696. 22901803 10.1016/j.cell.2012.07.018 PMC3431020

[14] Christofk HR, Vander Heiden MG, Harris MH, Ramanathan A, Gerszten RE, et al (2008) The M2 splice isoform of pyruvate kinase is important for cancer metabolism and tumour growth. Nature 452: 230–233. 18337823 10.1038/nature06734

[15] Kondo S, Lu Y, Debbas M, Lin AW, Sarosi I, et al (2003) Characterization of cells and gene-targeted mice deficient for the p53-binding kinase homeodomain-interacting protein kinase 1 (HIPK1). Proc Natl Acad Sci U S A 100: 5431–5436. 12702766 10.1073/pnas.0530308100 PMC154362

[16] Lee D, Park SJ, Sung KS, Park J, Lee SB, et al (2012) Mdm2 associates with Ras effector NORE1 to induce the degradation of oncoprotein HIPK1. EMBO Rep 13: 163–169. 22173032 10.1038/embor.2011.235 PMC3271333

[17] Zhao L, Wang H, Liu C, Liu Y, Wang X, et al (2010) Promotion of colorectal cancer growth and metastasis by the LIM and SH3 domain protein 1. Gut 59: 1226–1235. 20660701 10.1136/gut.2009.202739

[18] Grunewald TG, Kammerer U, Winkler C, Schindler D, Sickmann A, et al (2007) Overexpression of LASP-1 mediates migration and proliferation of human ovarian cancer cells and influences zyxin localisation. Br J Cancer 96: 296–305. 17211471 10.1038/sj.bjc.6603545 PMC2359999

[19] Zhang Y, Ye Y, Shen D, Jiang K, Zhang H, et al (2010) Identification of transgelin-2 as a biomarker of colorectal cancer by laser capture microdissection and quantitative proteome analysis. Cancer Sci 101: 523–529. 19930159 10.1111/j.1349-7006.2009.01424.x PMC11159707

[20] Morohashi Y, Balklava Z, Ball M, Hughes H, Lowe M (2010) Phosphorylation and membrane dissociation of the ARF exchange factor GBF1 in mitosis. Biochem J 427: 401–412. 20175751 10.1042/BJ20091681

[21] Miyasaka KY, Kida YS, Sato T, Minami M, Ogura T, et al (2007) Csrp1 regulates dynamic cell movements of the mesendoderm and cardiac mesoderm through interactions with Dishevelled and Diversin. Proc Natl Acad Sci U S A 104: 11274–11279. 17592114 10.1073/pnas.0702000104 PMC2040889

[22] Hirasawa Y, Arai M, Imazeki F, Tada M, Mikata R, et al (2006) Methylation status of genes upregulated by demethylating agent 5-aza-2′-deoxycytidine in hepatocellular carcinoma. Oncology 71: 77–85. 17341888 10.1159/000100475

[23] Goicoechea SM, Bednarski B, García-Mata R, Prentice-Dunn H, Kim HJ, et al (2009) Palladin contributes to invasive motility in human breast cancer cells. Oncogene 28: 587–598. 18978809 10.1038/onc.2008.408 PMC2633435

[24] Weitzdoerfer R, Fountoulakis M, Lubec G (2001) Aberrant expression of dihydropyrimidinase related proteins-2,-3 and -4 in fetal Down syndrome brain. J Neural Transm Suppl 61: 95–107. 10.1007/978-3-7091-6262-0_8 11771764

[25] Jung CR, Lim JH, Choi Y, Kim DG, Kang KJ, et al (2010) Enigma negatively regulates p53 through MDM2 and promotes tumor cell survival in mice. J Clin Invest 120: 4493–4506. 21060154 10.1172/JCI42674 PMC2993588

[26] Haynes J, Srivastava J, Madson N, Wittmann T, Barber DL (2011) Dynamic actin remodeling during epithelial-mesenchymal transition depends on increased moesin expression. Mol Biol Cell 22: 4750–4764. 22031288 10.1091/mbc.E11-02-0119 PMC3237619

[27] Yonezawa S, Higashi M, Yamada N, Yokoyama S, Kitamoto S, et al (2011) Mucins in human neoplasms: clinical pathology, gene expression and diagnostic application. Pathol Int 61: 697–716. 22126377 10.1111/j.1440-1827.2011.02734.x

[28] Wei X, Xu H, Kufe D (2007) Human mucin 1 oncoprotein represses transcription of the p53 tumor suppressor gene. Cancer Res 67: 1853–1858. 17308127 10.1158/0008-5472.CAN-06-3063

[29] Ren J, Li Y, Kufe D (2002) Protein kinase C delta regulates function of the DF3/MUC1 carcinoma antigen in beta-catenin signaling. J Biol Chem 277: 17616–17622. 11877440 10.1074/jbc.M200436200

[30] Schwappacher R, Rangaswami H, Su-Yuo J, Hassad A, Spitler R, et al (2013) cGMP-dependent protein kinase Iβ regulates breast cancer cell migration and invasion via a novel interaction with the actin/myosin-associated protein caldesmon. J Cell Sci 126: 1626–1636. 23418348 10.1242/jcs.118190 PMC3647439

[31] Mayanagi T, Morita T, Hayashi K, Fukumoto K, Sobue K, et al (2008) Glucocorticoid receptor-mediated expression of caldesmon regulates cell migration via the reorganization of the actin cytoskeleton. J Biol Chem 283: 31183–31196. 18772142 10.1074/jbc.M801606200 PMC2662183

[32] Rinaldo C, Siepi F, Prodosmo A, Soddu S (2008) HIPKs: Jack of all trades in basic nuclear activities. Biochim Biophys Acta 1783: 2124–2129. 18606197 10.1016/j.bbamcr.2008.06.006

[33] Rinaldo C, Prodosmo A, Siepi F, Soddu S (2007) HIPK2: a multitalented partner for transcription factors in DNA damage response and development. Biochem Cell Biol 85: 411–418. 17713576 10.1139/O07-071

[34] Berber S, Llamosas E, Thaivalappil P, Boag PR, Crossley M, et al (2013) Homeodomain interacting protein kinase (HPK-1) is required in the soma for robust germline proliferation in C. elegans. Dev Dyn 242: 1250–1261. 23904186 10.1002/dvdy.24023

[35] Burridge K, Chrzanowska-Wodnicka M (1996) Focal adhesions, contractility, and signaling. Annu Rev Cell Dev Biol 12: 463–518. 8970735 10.1146/annurev.cellbio.12.1.463

[36] Kim DH, Wirtz D (2013) Focal adhesion size uniquely predicts cell migration. FASEB J 27: 1351–1361. 23254340 10.1096/fj.12-220160 PMC3606534

[37] Ridley AJ, Schwartz MA, Burridge K, Firtel RA, Ginsberg MH, et al (2003) Cell migration: integrating signals from front to back. Science 302: 1704–1709. 14657486 10.1126/science.1092053

[38] Fu L, Qin YR, Xie D, Chow HY, Ngai SM, et al (2007) Identification of alpha-actinin 4 and 67 kDa laminin receptor as stage-specific markers in esophageal cancer via proteomic approaches. Cancer 110: 2672–2681. 17960614 10.1002/cncr.23110

[39] Kelly KA, Bardeesy N, Anbazhagan R, Gurumurthy S, Berger J, et al (2008) Targeted nanoparticles for imaging incipient pancreatic ductal adenocarcinoma. PLoS Med 5: e85. 18416599 10.1371/journal.pmed.0050085 PMC2292750

[40] Reynolds AB, Roczniak-Ferguson A (2004) Emerging roles for p120-catenin in cell adhesion and cancer. Oncogene 23: 7947–7956. 15489912 10.1038/sj.onc.1208161

[41] Shin DH, Chun YS, Lee KH, Shin HW, Park JW (2009) Arrest defective-1 controls tumor cell behaviour by acetylating myosin light chain kinase. PLoS One. 2009 4: e7451. 10.1371/journal.pone.0007451 PMC2758594 19826488

[42] Zhang J, Park SI, Artime MC, Summy JM, Shah AN, et al (2007) AFAP-110 is overexpressed in prostate cancer and contributes to tumorigenic growth by regulating focal contacts. J Clin Invest 117: 2962–2973. 17885682 10.1172/JCI30710 PMC1978423

[43] Iden S, Misselwitz S, Peddibhotla SS, Tuncay H, Rehder D, et al (2012) aPKC phosphorylates JAM-A at Ser285 to promote cell contact maturation and tight junction formation. J Cell Biol 196: 623–639. 22371556 10.1083/jcb.201104143 PMC3307692

[44] Cook DR, Rossman KL, Der CJ (2013) Rho guanine nucleotide exchange factors: regulators of Rho GTPase activity in development and disease. Oncogene In press. 10.1038/onc.2013.362 PMC4875565 24037532

[45] Germain EC, Santos TM, Rabinovitz I (2009) Phosphorylation of a novel site on the {beta} 4 integrin at the trailing edge of migrating cells promotes hemidesmosome disassembly. Mol Biol Cell 20: 56–67. 19005215 10.1091/mbc.E08-06-0646 PMC2613111

[46] Dans M, Gagnoux-Palacios L, Blaikie P, Klein S, Mariotti A, et al (2001) Tyrosine phosphorylation of the beta 4 integrin cytoplasmic domain mediates Shc signaling to extracellular signal-regulated kinase and antagonizes formation of hemidesmosomes. J Biol Chem 276: 1494–1502. 11044453 10.1074/jbc.M008663200

[47] Ji H, Wang J, Nika H, Hawke D, Keezer S, et al (2009) EGF-induced ERK activation promotes CK2-mediated disassociation of alpha-Catenin from beta-Catenin and transactivation of beta-Catenin. Mol Cell 36: 547–559. 19941816 10.1016/j.molcel.2009.09.034 PMC2784926

[48] Rivera AL, Pelloski CE, Gilbert MR, Colman H, De La Cruz C, et al (2010) MGMT promoter methylation is predictive of response to radiotherapy and prognostic in the absence of adjuvant alkylating chemotherapy for glioblastoma. Neuro Oncol 12: 116–121. 20150378 10.1093/neuonc/nop020 PMC2940581

[49] Yeo MG, Oh HJ, Cho HS, Chun JS, Marcantonio EE, et al (2011) Phosphorylation of Ser 21 in Fyn regulates its kinase activity, focal adhesion targeting, and is required for cell migration. J Cell Physiol 226: 236–247. 20658524 10.1002/jcp.22335

[50] Schramek H, Schumacher M, Wilflingseder D, Oberleithner H, Pfaller W (1997) Differential expression and activation of MAP kinases in dedifferentiated MDCK-focus cells. Am J Physiol 272: C383–C391. 9124279 10.1152/ajpcell.1997.272.2.C383

[51] Bellacosa A, Chan TO, Ahmed NN, Datta K, Malstrom S, et al (1998) Akt activation by growth factors is a multiple-step process: the role of the PH domain. Oncogene 17: 313–325. 9690513 10.1038/sj.onc.1201947

[52] Di Maira G, Salvi M, Arrigoni G, Marin O, Sarno S, et al (2005) Protein kinase CK2 phosphorylates and upregulates Akt/PKB. Cell Death Differ 12: 668–677. 15818404 10.1038/sj.cdd.4401604

[53] Kotliarova S, Pastorino S, Kovell LC, Kotliarov Y, Song H, et al (2008) Glycogen synthase kinase-3 inhibition induces glioma cell death through c-MYC, nuclear factor-kappaB, and glucose regulation. Cancer Res 68: 6643–6651. 18701488 10.1158/0008-5472.CAN-08-0850 PMC2585745

[54] Wang H, Chang-Wong T, Tang HY, Speicher DW (2010) Comparison of Extensive Protein Fractionation and Repetitive LC-MS/MS Analyses on Depth of Analysis for Complex Proteomes. J Proteome Res 9: 1032–1040. 20014860 10.1021/pr900927y PMC2870931

