# Correction: Quantification of Pancreatic Cancer Proteome and Phosphorylome: Indicates Molecular Events Likely Contributing to Cancer and Activity of Drug Targets

**DOI:** 10.1371/journal.pone.0107077

**Published:** 2014-08-25

**Authors:** The PLOS ONE Staff

## Notice of Republication

This article was republished on August 11, 2014, to replace incorrectly changed characters in the byline and Competing Interests statement. The publisher apologizes for the errors. Please download this article again to view the correct version. The originally published, uncorrected article and the republished, corrected article are provided here for reference.

## Supporting Information

File S1 Originally published, uncorrected article.
(PDF)

File S2 Republished, corrected article.
(PDF)
