# Supplementary material for: Quantification of Pancreatic Cancer Proteome and Phosphorylome: Indicates Molecular Events Likely Contributing to Cancer and Activity of Drug Targets
Source: PLoS One. 2014 Mar 26;9(3):e90948. doi: 10.1371/journal.pone.0090948 (PMC3966770; doi:10.1371/journal.pone.0090948)
Supplement: Methods S1 — This document contains supplemental methods. (DOCX) [file pone.0090948.s012.docx]

**Supplemental Methods**

**Frozen Clinical Tissue.** Ethical aspects and research protocol were approved by the BioBank Committee of the Institute of Liver Studies, King's College Hospital. Twelve cases of pancreatic head ductal adenocarcinoma were selected in the database of BioBank at the Institute of Liver Studies (Table S1, in Supplemental Tables S1). Initially cases 2 and 3 were selected but later found to have too little protein for this workflow. Therefore two additional cases were selected (Cases 13 and 14) to increase the number back to twelve. Small pieces of tissue were snap frozen from Whipple’s specimens and stored in a BioBank freezer. This process of tissue sampling was completed within 30 min. Paired samples of cancer (tumor) and background (non-tumor) were used for each case. Table S2&S3 (Supplemental Tables S1) describes tumor grade, whether recurrence was present, and other non-confidential clinical information.

**Tissue cell lysis.** Frozen clinical tissue samples were pulverized then ground into a fine powder using a Pestle and Mortar in the presence of liquid nitrogen. The powder was then transferred to eppendorf tubes containing 1.3 mL of ice cold lysis buffer (8M urea, 75 mM NaCl, 50 mM Tris-pH 8.2, one tablet of protease inhibitors cocktail (complete mini, Roche) per 10 mL of lysis buffer, and one tablet of phosphatase inhibitor cocktail (Roche) per 10 mL of lysis buffer). Samples were then sonicated at 20% Amplitude for 20 x 1 second, pulsing on and off, on ice (4°C). Following centrifugation at 12,500g for 10 min at 4^o^C, the protein concentration of each sample were then determined using the Bradford protein assay and microplate luminometer. Protein amounts used for this workflow for each TMT 8-plex are shown on Table S4, in Supplemental Tables S1.

**In-Solution Trypsin Digestion.**

Reduction, alkylation of cysteines, and digestion was performed on each lysate by following the Villén and Gygi, Nature Protocol, approach [Villén, J., Gygi S. The SCX/IMAC enrichment approach for global phosphorylation analysis by mass spectrometry. Nature Protocols. **3**, 1630 (2008)]. The digested samples were spun for 10 minutes at 2,500g and de-salted on 100mg SepPak tC18 cartridges (Waters, Milford, MA, USA). Peptides were eluted with 50% ACN/0.1% TFA and lyophilised.

**TMT Labelling.** Digested peptides from all samples were separately re-suspended in 200mM TEAB/10%ACN, mixed with their respective TMT 8-plex reagent (15mM final concentration), labelling plan shown in Table S5, in Supplemental Tables S1, and left to incubate for 1 hour at room temperature. The TMT reactions were then terminated with 0.25% hydroxylamine for 15 minutes. Samples were pooled into three TMT 8-plex and left to incubate for another 15 minutes. Each TMT 8-plex sample were acidified and the acentonitrile concentration diluted to below 5%, then divided into three aliquots (as shown in Table S6, Supplemental Tables S1) each of which were desalted on a 200mg SepPak tC18 cartridge, eluted, then lyophilized.

**SCX-HPLC.**

All 9 aliquots of lyophilized peptides (Table S6, Supplemental Tables S1) were re-suspended in SCX buffer C, then separated into 12 fractions by SCX-HPLC. The fractionation was carried out using a polySULFOETHYL-A column (PolyLC) and our SCX HPLC system (Waters Alliance 2695) according to the Villén and Gygi, Nature Protocol26, approach.

Buffer A: 0.1% TFA in water.

Buffer C: 7 mM KH2PO4, pH 2.65, 30% ACN (vol/vol).

Buffer D: 7 mM KH2PO4, 350 mM KCl, pH 2.65, 30% ACN (vol/vol).

**Immobilized Metal-Affinity Chromatography (IMAC) and TiO_2_.** Phosphopeptides were enriched by IMAC (Thermo Scientific Pierce product code 88300) or TiO_2_ (Thermo Scientific Pierce product code 88301), in accordance with manufacturer’s instructions.

**Graphite Spin Columns.** Following phosphopeptide enrichment, peptides were purified using graphite spin columns (Thermo Scientific Pierce product code 88302), according to manufacturer’s instructions.

**Liquid Chromatography – Tandem Mass Spectrometry (LC-MS/MS).** Peptides from all phospho-enriched fractions were re-suspended in 35 µl of 2% ACN/0.1% FA, then 8µL of each sample were injected onto a 0.1 × 20 mm pre-column self-packed with ReproSil C18, 5 µm (Dr. Maisch), using the Thermo Scientific Proxeon EASY-nLC II system. Non-enriched fractions were re-suspended in 1.5 mL 2%ACN/0.1% FA, further diluted 1 in 5, then 5µL injection. Peptides were then resolved using an increasing gradient of 0.1% formic acid in acetonitirile (10 to 25% over 90 minutes) through a 0.075 × 150 mm self-packed column with ReproSil C18, 3 µm (Dr. Maisch) at a flow rate of 300nL/min. Mass spectra were acquired on a Thermo Scientific LTQ Orbitrap Velos throughout the chromatographic run (115 minutes), using 10 higher collision induced dissociation (HCD) FTMS scans at 15000 resolving power @ 400 *m/z*, following each FTMS scan (2 x µScans at 30000 resolving power @ 400 *m/z*). HCD was carried out on 10 of the most intense ions from each FTMS scan then put on a dynamic exclusion list for 30secs (10 ppm *m/z* window). AGC ion injection target for each FTMS scan were 1000000 (500ms max injection time). AGC ion injection target for each HCD FTMS2 scan were 50000 (500ms max ion injection time). Each sample were analysed by three LC-MS/MS analytical repeats, where the third analytical repeat used a time dependent rejection list, rejecting all peptide ions that were identified as peptides, with 1%FDR, in one of the first two analytical repeats.

**Peptide identification and quantification**.

*Proteome Discoverer*

In total there were 324 Raw data files (3 x TMT 8-plex sample X3 aliquots X12 fractions X3 analytical repeats), where there were 108 raw data files belonging to each TMT 8-plex. All 108 raw data files from the first TMT 8-plex sample were combined for a Mudpit search using Proteome Discoverer, as described below. This was also carried out for the second and third TMT 8-plex samples.

Raw data were submitted to the Thermo Scientific Proteome Discoverer 1.3 software, using the Spectrum Files node. Spectrum selector was set to its default values, while the Mascot node, was set up to search data against the uniprot_sprot database, taxonomy *homo sapiens*. This node was programmed to search for tryptic peptides (two missed cleavages) with static modifications of carbamidomethyl (C), TMT 6-plex (K), and TMT 6-plex (N-Term). Dynamic modifications were set to deamidation (N/Q), oxidation (M), and phosphorylation of STY. Precursor mass tolerance was set to 20ppm and fragment (b and y ions) mass tolerance to 20mmu. Spectra were also searched against SEQUEST, using the same database, modifications, and tolerances as the Mascot node. Spectra were also search using the PhosphoRS2.0 (fragment mass tolerance of 20mmu, considering neutral loss peaks for CID and HCD) and Percolator nodes. More information about the PhosphoRS node can be obtained in the paper; *Taus T, et al. Universal and confident phosphorylation site localization using phosphoRS. J Proteome Res. 2011 Dec 2;10(12):5354-62*.

The reporter ions quantifier node was set up to measure the raw intensity values of TMT 8-plex mono-isotopic ions, from all identified PSMs, at; 126.12773 *m/z* (126), 127.12476 *m/z* (127e), 127.13108 *m/z* (127), 128.13444 *m/z* (128), 129.13147 *m/z* (129e), 129.13779 *m/z* (129), 130.14115 *m/z* (130), 131.13818 *m/z* (131), using a tolerance of 20ppm after centroiding. No filters were applied at this stage using Proteome Discoverer, therefore all raw intensity values were exported to excel for later processing and filtering using in house software.

**Bioinformatics**

Statistical analysis was performed to investigate relevant regulations with respect to the disease group T (*pancreatic tumor tissue*) and matched control NT (*non-tumor tissue*) from 12 patients.

Accuracy and precision of mass spectrometry quantification approaches can suffer from issues such as Experimental bias, Systematic errors, Random Errors (Heterogeneity of Variance), and missing quantification values. To improve accuracy and precision we assessed the quality of our data, then filtered and normalised as described below.

*MS quality - Data Filtering and normalisation:*

All spectra which did not include signal intensities in all TMT 8-plex reporter channels were deleted. For normalisation sum-scaling was performed. Due to differences between samples it is advisable to normalize data before further processing. The effects of the normalization can be observed in Figure S1A (data not normalized) and Figure S1B (data normalized). After sum scaling the data is better zero centred.

*Statistics*

Log_2_ ratios are calculated, which show the regulations T over NT for all (median) and for each patient. For protein ratios all peptides which are not phosphorylated were used and combined with the median. For the phosphopeptide ratios all peptides which have a pRS probability over 75% in any phosphorylation position was used. A phosphopeptide T/NT log_2_ ratio is the median T/NT log_2_ ratio from all PSMs unique to that specific peptide sequence.

The ratio’s were calculated:

$${{{log}_{2}({}_{i}T}}/{{}_{i}{{NT}})}$$

*Where I = patient 1,4,5,6,7,8,9,10,11,12,13,14*

For the data analysis a one sided t-test (or one-sample [location test](http://en.wikipedia.org/wiki/Location_test)) will be used [http://en.wikipedia.org/wiki/T_test]. A one side t-test is able to detect significant regulation.

P-values and log_2_ ratios can be observed in File S4. Significant p-values were highlighted in red text and yellow background. Annotation with GO-terms, KEGG-pathways and Drugbank info were added at the protein level, and annotation from phosphosite plus were added at the phosphorylation site level.

*All XCalibur raw data files and Proteome Discoverer MSF files can be downloaded from BOX* [*www.box.com*](http://www.box.com) *(please request access from david.britton@proteomics.com).*

*PLS-DA:*

In order to investigate the multivariate dataset partial least squares discriminant analysis (PLS-DA) was performed on the data set that consisted of normalised isobaric tag intensities and log_2_ T/NT ratios of all phosphopeptides from the three arms of the workflow. PLS modelling is often used for such datasets with correlated input variables and several to many result variables. One specifies which variables in the dataset are the predictors (X), and which are the dependent variables (Y). The PLS model then finds the relation between the groups of variables. A PLS model is expressed as a set of X-score vectors, Y-score vectors, X-weight and Y-weight vectors, with PLS model dimensions. Each dimension (index a) expresses a linear relation between an X-score vector (ta) and Y-score vector (ua). The weight vectors of each model dimension express how the X-variables are combined to form ta, and the Y-variables are combined to form ua. In this way the data are modelled as a set of "factors" in X and Y and their relationships. Bi-Plots of the scores and weights facilitate the model interpretation. The biplot used to graph the PLS results can be used to interpret relationships between the three different enrichment arms of the workflow and PLS also detects severe outliers, which are observations in the component space whose distance to the centre of gravity of the data cloud is considered to be too high. In practice, severe outliers are detected by the statistic T2 Hotelling defined as the Mahalanobis distance between the observation projection onto the principal space and the centre of gravity of the data cloud. Hence, a given observation is “out of control” when a critical distance based on the 95% and 99% confidence level is reached. All PLS-DA computations were done using SIMCAP software package (version 11.5, UMETRICS).
