# Supplementary material for: Quantification of Pancreatic Cancer Proteome and Phosphorylome: Indicates Molecular Events Likely Contributing to Cancer and Activity of Drug Targets
Source: PLoS One. 2014 Mar 26;9(3):e90948. doi: 10.1371/journal.pone.0090948 (PMC3966770; doi:10.1371/journal.pone.0090948)
Supplement: Tables S1 — This document contains Tables S1–S8. Table S1, Fourteen cases of pancreatic head ductal adenocarcinoma were selected from Institute of Liver Studies BioBank. Specimens from cases 2 and 3 yielded low protein amounts during protein extraction therefore were omitted from the study. Table S2, Information on tumor stage and recurrence are shown here. Yellow cases showed recurrence between 2 & 31 months after tumor removal. The difference between stage IIA and IIB is only the presence or absence of lymph node metastasis. Table S3, Additional non-confidential clinical information about patient and tumor. Table S4, Protein amounts from each sample per TMT 8-plex, used for the SysQuant workflow in this study. Table S5, Peptides from each specimen are labelled with different tandem mass tags (TMT). All peptides from case 1 tumor, case 10 non-tumor, and case 11 tumor were labelled with the 126 Da tandem mass tag (TMT) while peptides from case 1 non-tumor, case 10 tumor and case 11 non-tumor were labelled with the lighter 127 Da tandem mass tag (TMT), and so on as shown below. The lighter 127 (127e) and heavier 127 only differ in mass by 6 milli-Daltons, as do 129e and 129 reporter ions. Table S6, Nine aliquots of TMT labelled peptides were separated by SCX-HPLC. Table S7, Accession numbers of proteins which yielded phosphopeptides demonstrating log2 T/NT ratios of ≥1, or ≤−1 (More than 2 fold up/down- regulated), were selected separately from each case. Accession numbers were then uploaded to the DAVID Bioinformatic resource (separately for each case) which identified KEGG signaling pathways matched with greatest significance based on p-values and Benjamini scores. KEGG pathways with Benjamini scores ≤0.05 were highlighted in Yellow. Table S8, Case by case – Here we selected all phosphopeptides displaying log2 T/NT ratios ≥1 or ≤−1, that also contain phosphorylation sites that are known to either induce activation or inhibition of the phosphorylated enzyme (based on PhosphoSit [file pone.0090948.s013.docx]

Table S1.

| Case # | Sample name | BioBank # | Tissue Type | Tissue weight (mg) |
| --- | --- | --- | --- | --- |
| 1 | 1T | 14981 | Pancreatic cancer | 362 |
| 1 | 1NT | 14980 | Background pancreas | 198 |
| *2* | *2T* | *14837* | *Pancreatic cancer* | *102* |
| *2* | *2NT* | *14836* | *Background pancreas* | *128* |
| *3* | *3T* | *14786* | *Pancreatic cancer* | *135* |
| *3* | *3NT* | *14785* | *Background pancreas* | *56* |
| 4 | 4T | 14938 | Pancreatic cancer | 458 |
| 4 | 4NT | 14987 | Background pancreas | 231 |
| 5 | 5T | 11967 | Pancreatic cancer | 204 |
| 5 | 5NT | 11966 | Background pancreas | 223 |
| 6 | 6T | 11946 | Pancreatic cancer | 204 |
| 6 | 6NT | 11945 | Background pancreas | 136 |
| 7 | 7T | 11250 | Pancreatic cancer | 303 |
| 7 | 7NT | 11251 | Background pancreas | 240 |
| 8 | 8T | 10652 | Pancreatic cancer | 315 |
| 8 | 8NT | 10653 | Background pancreas | 273 |
| 9 | 9T | 10619 | Pancreatic cancer | 247 |
| 9 | 9NT | 10618 | Background pancreas | 223 |
| 10 | 10T | 10666 | Pancreatic cancer | 436 |
| 10 | 10NT | 10665 | Background pancreas | 489 |
| 11 | 11T | 14894 | Pancreatic cancer | 145 |
| 11 | 11NT | 14896 | Background pancreas | 150 |
| 12 | 12T | 16195 | Pancreatic cancer | 113 |
| 12 | 12NT | 16194 | Background pancreas | 110 |
| 13 | 13T | 14784 | Pancreatic cancer | 202 |
| 13 | 13NT | 14783 | Background pancreas | 190 |
| 14 | 14T | 14950 | Pancreatic cancer | 190 |
| 14 | 14NT | 14949 | Background pancreas | 120 |

Table S2.

| 1 | pT3N1 | Stage IIB |
| --- | --- | --- |
| 4 | pT3N1 | Stage IIB |
| **5** | **pT3N0** | **Stage IIA** |
| 6 | pT3N1 | Stage IIB |
| 7 | pT3N1 | Stage IIB |
| **8** | **pT3N0** | **Stage IIA** |
| 9 | pT3N1 | Stage IIB |
| **10** | **pT3N0** | **Stage IIA** |
| 11 | pT3N1 | Stage IIB |
| 12 | pT3N1 | Stage IIB |
| 13 | pT3N1 | Stage IIB |
| 14 | pT3N1 | Stage IIB |

Table S3.

| **Case #** | **Recurrence** | **Lymph node mets** | **Sex** | **Age** | **Tissue in freezer (mo)** | **HbA1c (<6.0)** |
| --- | --- | --- | --- | --- | --- | --- |
| **1** | Yes, 11 mo | Yes | F | 74 | 7 | 5.8 |
| **4** | Yes, 10 mo | Yes | M | 70 | 13 | 7.5 |
| **5** | Yes, 19 mo | No | F | 61 | 18 | 9.6 |
| **6** | No (follow up 20 mo) | Yes | M | 67 | 19 | 6.5 |
| **7** | Yes, 16 mo | Yes | M | 79 | 20 | 6.6 |
| **8** | No (follow up 23 mo) | No | M | 80 | 21 | NA |
| **9** | Yes, 5 mo | Yes | M | 67 | 23 | 8.8 |
| **10** | Yes, 31 mo | No | F | 71 | 28 | 5.6 |
| **11** | Yes, 17 mo | Yes | M | 86 | 24 | 7.9 |
| **12** | Yes, 10 mo | Yes | M | 40 | 3 | 5.6 |
| **13** | Yes, 21 mo | Yes | M | 83 | 12 | 6.2 |
| **14** | Yes, 2 mo | Yes | M | 53 | 8 | 10.5 |

Table S4.

| Sample-Name | µg | µg per TMT 8-plex |
| --- | --- | --- |
| **1T** | 3900 | **31200 (TMT 8-plex-1)** |
| **1NT** | 3900 |  |
| **4T** | 3900 |  |
| **4NT** | 3900 |  |
| **5T** | 3900 |  |
| **5NT** | 3900 |  |
| **6T** | 3900 |  |
| **6NT** | 3900 |  |
| **10NT** | 3900 | **29800 (TMT 8-plex-2)** |
| **10T** | 3900 |  |
| **9NT** | 3900 |  |
| **9T** | 3900 |  |
| **8NT** | 3900 |  |
| **8T** | 3900 |  |
| **7NT** | 3200 |  |
| **7T** | 3200 |  |
| **11T** | 3900 | **25720 (TMT 8-plex-3)** |
| **11NT** | 3900 |  |
| **12T** | 2800 |  |
| **12NT** | 2800 |  |
| **13T** | 2560 |  |
| **13NT** | 2560 |  |
| **14T** | 3600 |  |
| **14NT** | 3600 |  |

Table S5.

| TMT reporter mass: | 126 | 127e | 127 | 128 | 129e | 129 | 130 | 131 |
| --- | --- | --- | --- | --- | --- | --- | --- | --- |
| TMT 8-plex-1 | 1T | 1NT | 4T | 4NT | 5T | 5NT | 6T | 6NT |
| TMT 8-plex-2 | 10NT | 10T | 9NT | 9T | 8NT | 8T | 7NT | 7T |
| TMT 8-plex-3 | 11T | 11NT | 12T | 12NT | 13T | 13NT | 14T | 14NT |

Table S6.

| Sample/aliquot | SCX-HPLX | Enrichment method |
| --- | --- | --- |
| TMT 8-plex-1a | 12 x fractions | for TiO_2_ |
| TMT 8-plex-1b | 12 x fractions | for IMAC |
| TMT 8-plex-1c | 12 x fractions | No enrichment |
| TMT 8-plex-2a | 12 x fractions | No enrichment |
| TMT 8-plex-2b | 12 x fractions | for IMAC |
| TMT 8-plex-2c | 12 x fractions | for TiO_2_ |
| TMT 8-plex-3a | 12 x fractions | for TiO_2_ |
| TMT 8-plex-3b | 12 x fractions | for IMAC |
| TMT 8-plex-3c | 12 x fractions | No enrichment |

Table S7.

| Case | Term | Count | % | **PValue** | Genes | List Total | Pop Hits | Pop Total | Fold Enrichment | Bonferroni | Benjamini | FDR |
| --- | --- | --- | --- | --- | --- | --- | --- | --- | --- | --- | --- | --- |
| T1/NT1 | hsa04530:Tight junction | 16 | 4.8338369 | **1.67E-07** | Q96A65, Q9H4G0, Q3MNF0, Q3MNF1, P35221, Q05655, P35222, P35579, O43491, P55196, Q9P2M7, P35580, Q7Z406, Q3MIV8, Q9Y624, P31749, P35749, Q14247, Q07157 | 113 | 134 | 5085 | 5.3731343 | 1.92E-05 | 1.92E-05 | 1.90E-04 |
| T1/NT1 | hsa04520:Adherens junction | 11 | 3.3232628 | **5.71E-06** | P55196, O43318, P29350, P18206, Q8WWI1, O60716, Q9UQB8, P06241, P35221, P35222, Q07157 | 113 | 77 | 5085 | 6.4285714 | 6.57E-04 | 3.28E-04 | 0.0065072 |
| T1/NT1 | hsa05130:Pathogenic Escherichia coli infection | 7 | 2.1148036 | **1.45E-03** | Q92974, P68366, Q9BQE3, P06241, P35222, P19338, Q14247 | 113 | 57 | 5085 | 5.5263158 | 0.1539067 | 5.42E-02 | 1.6417623 |
| T1/NT1 | hsa04660:T cell receptor signaling pathway | 9 | 2.7190332 | **2.44E-03** | O43318, P29350, O95999, O14920, Q9BXL7, Q14934, P06241, P36507, P31749 | 113 | 108 | 5085 | 3.75 | 0.2445643 | 6.77E-02 | 2.7397466 |
| T1/NT1 | hsa05416:Viral myocarditis | 7 | 2.1148036 | **4.47E-03** | P35580, Q3MNF0, Q7Z406, Q3MNF1, Q04637, P06241, Q3MIV8, P11532, P35749, P35579 | 113 | 71 | 5085 | 4.4366197 | 0.4025028 | 9.79E-02 | 4.9732348 |
| T1/NT1 | hsa04662:B cell receptor signaling pathway | 7 | 2.1148036 | **5.85E-03** | P29350, O95999, O14920, Q9BXL7, Q14934, P36507, P31749 | 113 | 75 | 5085 | 4.2 | 0.4908272 | 1.06E-01 | 6.4670057 |
| T1/NT1 | hsa05412:Arrhythmogenic right ventricular cardiomyopathy (ARVC) | 7 | 2.1148036 | **6.24E-03** | P02545, P15924, P35221, P17661, Q99959, P35222, P11532 | 113 | 76 | 5085 | 4.1447368 | 0.5132938 | 9.78E-02 | 6.8841508 |
| T1/NT1 | hsa04670:Leukocyte transendothelial migration | 8 | 2.4169184 | **1.48E-02** | P55196, P18206, O60716, P26038, Q96FS4, P35221, P35222, Q9Y624 | 113 | 118 | 5085 | 3.0508475 | 0.8191351 | 1.92E-01 | 15.580936 |
| T1/NT1 | hsa04370:VEGF signaling pathway | 6 | 1.8126888 | **2.41E-02** | P29474, Q14934, P36507, P04792, P47712, P31749 | 113 | 75 | 5085 | 3.6 | 0.9396232 | 2.68E-01 | 24.274084 |
| T1/NT1 | hsa05221:Acute myeloid leukemia | 5 | 1.510574 | **3.81E-02** | O14920, P36507, P42229, P31749, P29590 | 113 | 58 | 5085 | 3.8793103 | 0.9884985 | 3.60E-01 | 35.743558 |
| T4/NT4 | hsa04530:Tight junction | 13 | 4.5454545 | **1.18E-06** | Q3MNF1, Q9UDY2, P11171, Q05655, P35222, P35579, Q9P2M7, P55196, P35580, Q5JTD0, Q7Z406, Q9Y624, P31749, P35749 | 83 | 134 | 5085 | 5.9436252 | 1.15E-04 | 1.15E-04 | 0.0012992 |
| T4/NT4 | hsa04660:T cell receptor signaling pathway | 8 | 2.7972028 | **1.65E-03** | O43318, P29350, O14920, Q9BXL7, P28482, Q14934, O75791, P31749 | 83 | 108 | 5085 | 4.5381526 | 0.1491123 | 7.76E-02 | 1.8053893 |
| T4/NT4 | hsa04662:B cell receptor signaling pathway | 6 | 2.0979021 | **6.89E-03** | P29350, O14920, Q9BXL7, P28482, Q14934, P31749 | 83 | 75 | 5085 | 4.9012048 | 0.4920664 | 2.02E-01 | 7.358219 |
| T4/NT4 | hsa05412:Arrhythmogenic right ventricular cardiomyopathy (ARVC) | 6 | 2.0979021 | **7.28E-03** | P02545, P15924, P17661, Q99959, P35222, P11532 | 83 | 76 | 5085 | 4.8367153 | 0.5114174 | 1.64E-01 | 7.76333 |
| T4/NT4 | hsa04270:Vascular smooth muscle contraction | 7 | 2.4475524 | **8.99E-03** | O43306, Q05682, Q3MNF1, P28482, Q05655, O14974, P35749, Q96A00 | 83 | 112 | 5085 | 3.8290663 | 0.5873256 | 1.62E-01 | 9.5038909 |
| T4/NT4 | hsa05221:Acute myeloid leukemia | 5 | 1.7482517 | **1.38E-02** | O14920, P28482, Q01196, P40763, P31749 | 83 | 58 | 5085 | 5.2814707 | 0.7432498 | 2.03E-01 | 14.221884 |
| T4/NT4 | hsa04722:Neurotrophin signaling pathway | 7 | 2.4475524 | **1.44E-02** | O14920, P28482, Q05655, Q13554, Q99759, P31749, Q99523 | 83 | 124 | 5085 | 3.4585115 | 0.7589093 | 1.84E-01 | 14.828775 |
| T4/NT4 | hsa04910:Insulin signaling pathway | 7 | 2.4475524 | **2.11E-02** | O14920, Q15642, P28482, P13861, P46019, P31749, P10644 | 83 | 135 | 5085 | 3.1767068 | 0.8762019 | 2.30E-01 | 20.998908 |
| T4/NT4 | hsa04010:MAPK signaling pathway | 10 | 3.4965035 | **2.70E-02** | Q14315, O43318, Q01201, O14920, P28482, Q14934, P21333, P11831, Q99759, P31749 | 83 | 267 | 5085 | 2.2945715 | 0.9314064 | 2.57E-01 | 26.090432 |
| T4/NT4 | hsa05416:Viral myocarditis | 5 | 1.7482517 | **2.70E-02** | P35580, Q7Z406, Q3MNF1, P11532, P35749, P35579 | 83 | 71 | 5085 | 4.3144409 | 0.9314481 | 2.35E-01 | 26.095508 |
| T4/NT4 | hsa04370:VEGF signaling pathway | 5 | 1.7482517 | **3.22E-02** | P49023, P29474, P28482, Q14934, P31749 | 83 | 75 | 5085 | 4.0843373 | 0.9594031 | 2.53E-01 | 30.337414 |
| T4/NT4 | hsa04520:Adherens junction | 5 | 1.7482517 | **3.50E-02** | P55196, O43318, P29350, P28482, P35222 | 83 | 77 | 5085 | 3.9782507 | 0.9694356 | 2.52E-01 | 32.533139 |
| T4/NT4 | hsa04510:Focal adhesion | 8 | 2.7972028 | **4.23E-02** | Q14315, P49023, P28482, P02452, P21333, P35222, O14974, P31749 | 83 | 201 | 5085 | 2.4384104 | 0.9855755 | 2.78E-01 | 38.013707 |
| T5/NT5 | hsa04270:Vascular smooth muscle contraction | 11 | 3.3742331 | **8.26E-05** | Q14573, P63092, Q3MNF0, Q3MNF1, P10398, Q96A00, Q9Y6F6, O43306, Q15746, P28482, P36507, Q3MIV8, P35749, O15085 | 105 | 112 | 5085 | 4.7563776 | 0.0088823 | 8.88E-03 | 0.0929764 |
| T5/NT5 | hsa04530:Tight junction | 10 | 3.0674847 | **1.55E-03** | Q9H4G0, B7Z7H6, Q3MNF0, Q3MNF1, O95049, P35222, P35579, Q9P2M7, P35580, Q7Z406, Q3MIV8, P35749, Q07157 | 105 | 134 | 5085 | 3.6140725 | 0.1538314 | 8.01E-02 | 1.7264351 |
| T5/NT5 | hsa04540:Gap junction | 8 | 2.4539877 | **2.13E-03** | O43306, Q14573, P63092, P68366, Q9BQE3, P28482, P36507, Q07157 | 105 | 89 | 5085 | 4.35313 | 0.2057072 | 7.39E-02 | 2.3725258 |
| T5/NT5 | hsa04510:Focal adhesion | 12 | 3.6809816 | **2.48E-03** | Q14315, P22105, P18206, Q15746, Q9Y490, Q92934, P28482, P02452, P21333, Q15942, B2ZZ83, P35222 | 105 | 201 | 5085 | 2.891258 | 0.235444 | 6.49E-02 | 2.7601375 |
| T5/NT5 | hsa05416:Viral myocarditis | 7 | 2.1472393 | **3.09E-03** | O43432, P35580, Q3MNF0, Q7Z406, Q3MNF1, Q04637, Q3MIV8, P11532, P35749, P35579 | 105 | 71 | 5085 | 4.7746479 | 0.2843357 | 6.47E-02 | 3.427805 |
| T5/NT5 | hsa05130:Pathogenic Escherichia coli infection | 6 | 1.8404908 | **5.86E-03** | Q92974, P68366, P05783, Q9BQE3, P35222, P19338 | 105 | 57 | 5085 | 5.0977444 | 0.4696496 | 1.00E-01 | 6.398432 |
| T5/NT5 | hsa05221:Acute myeloid leukemia | 6 | 1.8404908 | **6.31E-03** | Q92934, P28482, P10398, P36507, P40763, P29590 | 105 | 58 | 5085 | 5.0098522 | 0.4949715 | 9.30E-02 | 6.8746491 |
| T5/NT5 | hsa04720:Long-term potentiation | 6 | 1.8404908 | **1.22E-02** | Q14573, Q13522, P28482, P10398, Q13554, P36507 | 105 | 68 | 5085 | 4.2731092 | 0.7349611 | 1.53E-01 | 12.928771 |
| T5/NT5 | hsa04520:Adherens junction | 6 | 1.8404908 | **2.01E-02** | P29350, P18206, O60716, P28482, P35222, Q07157 | 105 | 77 | 5085 | 3.7736549 | 0.8879411 | 2.16E-01 | 20.403138 |
| T5/NT5 | hsa05216:Thyroid cancer | 4 | 1.2269939 | **2.07E-02** | P28482, P36507, P35222, Q16204 | 105 | 29 | 5085 | 6.679803 | 0.8949838 | 2.02E-01 | 20.93999 |
| T5/NT5 | hsa05213:Endometrial cancer | 5 | 1.5337423 | **2.11E-02** | Q92934, P28482, P10398, P36507, P35222 | 105 | 52 | 5085 | 4.6565934 | 0.8999753 | 1.89E-01 | 21.340372 |
| T5/NT5 | hsa05110:Vibrio cholerae infection | 5 | 1.5337423 | **2.69E-02** | P13569, P63092, O43731, P60468, Q07157 | 105 | 56 | 5085 | 4.3239796 | 0.9474237 | 2.18E-01 | 26.441898 |
| T5/NT5 | hsa04912:GnRH signaling pathway | 6 | 1.8404908 | **4.94E-02** | O43306, Q14573, P63092, P28482, Q13554, P36507 | 105 | 98 | 5085 | 2.9650146 | 0.9958125 | 3.44E-01 | 43.497593 |
| T6/NT6 | hsa04530:Tight junction | 11 | 4.0892193 | **1.97E-05** | P55196, P35580, Q9H4G0, Q7Z406, Q5JTD0, P35221, P35749, P35579, Q9Y624, P31749, Q14247 | 75 | 134 | 5085 | 5.5656716 | 0.001774 | 1.77E-03 | 0.0214598 |
| T6/NT6 | hsa04910:Insulin signaling pathway | 8 | 2.9739777 | **3.25E-03** | Q92934, Q14432, O95685, P10398, P36507, P13861, P31749, P10644 | 75 | 135 | 5085 | 4.0177778 | 0.2542474 | 1.36E-01 | 3.483869 |
| T6/NT6 | hsa05213:Endometrial cancer | 5 | 1.8587361 | **6.60E-03** | Q92934, P35221, P10398, P36507, P31749 | 75 | 52 | 5085 | 6.5192308 | 0.4491342 | 1.80E-01 | 6.9537378 |
| T6/NT6 | hsa04670:Leukocyte transendothelial migration | 7 | 2.6022305 | **7.05E-03** | P55196, P18206, O60716, Q96FS4, P35221, Q9NRY4, Q9Y624 | 75 | 118 | 5085 | 4.0220339 | 0.4709377 | 1.47E-01 | 7.4068415 |
| T6/NT6 | hsa05221:Acute myeloid leukemia | 5 | 1.8587361 | **9.69E-03** | Q92934, Q01196, P10398, P36507, P31749 | 75 | 58 | 5085 | 5.8448276 | 0.5838409 | 1.61E-01 | 10.054798 |
| T6/NT6 | hsa05416:Viral myocarditis | 5 | 1.8587361 | **1.93E-02** | P35580, Q7Z406, P11532, P35749, P35579 | 75 | 71 | 5085 | 4.7746479 | 0.8265815 | 2.53E-01 | 19.085621 |
| T6/NT6 | hsa05220:Chronic myeloid leukemia | 5 | 1.8587361 | **2.31E-02** | Q92934, Q01196, P10398, P36507, P31749 | 75 | 75 | 5085 | 4.52 | 0.8779095 | 2.59E-01 | 22.446264 |
| T6/NT6 | hsa04520:Adherens junction | 5 | 1.8587361 | **2.52E-02** | P55196, O43318, P18206, O60716, P35221 | 75 | 77 | 5085 | 4.4025974 | 0.8991223 | 2.49E-01 | 24.214903 |
| T6/NT6 | hsa04810:Regulation of actin cytoskeleton | 8 | 2.9739777 | **3.58E-02** | P35580, P18206, Q7Z406, P53667, P10398, Q9NRY4, P36507, P35579 | 75 | 215 | 5085 | 2.5227907 | 0.9623427 | 3.05E-01 | 32.724673 |
| T6/NT6 | hsa05223:Non-small cell lung cancer | 4 | 1.4869888 | **4.32E-02** | Q92934, P10398, P36507, P31749 | 75 | 54 | 5085 | 5.0222222 | 0.9812867 | 3.28E-01 | 38.177535 |
| T7/NT7 | hsa04520:Adherens junction | 9 | 3.8793103 | **9.44E-06** | P55196, P08069, P29350, P18206, Q8WWI1, O60716, P06241, P18031, Q07157 | 72 | 77 | 5085 | 8.2548701 | 8.77E-04 | 8.77E-04 | 0.0103315 |
| T7/NT7 | hsa04510:Focal adhesion | 10 | 4.3103448 | **1.75E-03** | P08069, P18206, Q9Y490, P49841, P49023, P02452, P21333, P06241, Q15942, P31749 | 72 | 201 | 5085 | 3.5136816 | 0.150061 | 7.81E-02 | 1.8957344 |
| T7/NT7 | hsa04530:Tight junction | 8 | 3.4482759 | **2.46E-03** | P55196, Q9H4G0, Q7Z406, O95049, P35749, P31749, Q14247, Q07157 | 72 | 134 | 5085 | 4.2164179 | 0.2049008 | 7.36E-02 | 2.6629666 |
| T8/NT8 | hsa04530:Tight junction | 20 | 2.8129395 | **2.15E-06** | Q9H4G0, Q96A65, O95049, Q9UDY2, P11171, Q05655, P35579, Q13813, Q8TEW0, P55196, Q9P2M7, P35580, Q5JTD0, Q7Z406, Q9Y2J2, Q9Y624, P31749, P35749, Q07157, Q14247 | 213 | 134 | 5085 | 3.5631701 | 2.84E-04 | 2.84E-04 | 0.0025155 |
| T8/NT8 | hsa03040:Spliceosome | 18 | 2.5316456 | **1.49E-05** | P52272, P26368, O75643, P49756, O60231, Q13573, Q13247, Q07955, Q9UKV3, Q08170, Q9Y5S9, P08621, Q13242, Q00839, P61978, P09651, Q15365, P38159 | 213 | 126 | 5085 | 3.4104628 | 0.0019674 | 9.84E-04 | 0.0174202 |
| T8/NT8 | hsa04270:Vascular smooth muscle contraction | 15 | 2.1097046 | **1.99E-04** | Q05655, O60237, Q9NZN5, O14974, P47712, Q96A00, Q9Y6F6, Q15746, Q05682, P47901, P28482, P36507, P22694, O15085, P35749 | 213 | 112 | 5085 | 3.1973089 | 0.0259813 | 8.74E-03 | 0.2326065 |
| T8/NT8 | hsa04520:Adherens junction | 11 | 1.5471167 | **1.22E-03** | P55196, O43318, P29350, P18206, Q8WWI1, O60716, P28482, Q9UQB8, P18031, Q8TEW0, Q07157 | 213 | 77 | 5085 | 3.4104628 | 0.148375 | 3.94E-02 | 1.4107526 |
| T8/NT8 | hsa04910:Insulin signaling pathway | 15 | 2.1097046 | **1.35E-03** | P49841, Q92934, Q15642, O95685, P62753, P35568, P13861, P18031, Q9Y4H2, P54646, P28482, P36507, P22694, P31749, P46019 | 213 | 135 | 5085 | 2.6525822 | 0.1636104 | 3.51E-02 | 1.5680658 |
| T8/NT8 | hsa04660:T cell receptor signaling pathway | 13 | 1.8284107 | **1.64E-03** | O95999, P49841, Q13177, O75791, O15111, O43318, P05412, P29350, Q14934, P28482, Q16539, P36507, P31749 | 213 | 108 | 5085 | 2.8736307 | 0.1947729 | 3.55E-02 | 1.8981408 |
| T8/NT8 | hsa05220:Chronic myeloid leukemia | 10 | 1.4064698 | **3.68E-03** | Q13547, Q9UQC2, Q92934, Q92769, P28482, P36507, P46527, P42229, P31749, O15111 | 213 | 75 | 5085 | 3.1830986 | 0.385359 | 6.72E-02 | 4.2142762 |
| T8/NT8 | hsa04662:B cell receptor signaling pathway | 10 | 1.4064698 | **3.68E-03** | P05412, P29350, O95999, P49841, P28482, Q14934, P36507, P31749, O15111, P07948 | 213 | 75 | 5085 | 3.1830986 | 0.385359 | 6.72E-02 | 4.2142762 |
| T8/NT8 | hsa04010:MAPK signaling pathway | 21 | 2.9535865 | **7.33E-03** | P10636, P21333, P15336, Q13177, P47712, O15111, O95819, Q14315, Q9UBI6, O43318, P05412, P16949, P28482, Q14934, Q16539, P36507, Q9NYJ8, P04792, P22694, Q99759, P31749 | 213 | 267 | 5085 | 1.8776705 | 0.621331 | 1.14E-01 | 8.2319478 |
| T8/NT8 | hsa04810:Regulation of actin cytoskeleton | 18 | 2.5316456 | **7.81E-03** | P02751, P18206, P16144, Q9UQB8, P19634, Q13177, Q9NZN5, O14974, P35579, P35580, Q9UBI6, P15311, Q15746, Q7Z406, Q9Y2X7, P28482, P36507, Q13576 | 213 | 215 | 5085 | 1.9986898 | 0.6449799 | 1.09E-01 | 8.7539777 |
| T8/NT8 | hsa05221:Acute myeloid leukemia | 8 | 1.1251758 | **9.77E-03** | Q92934, P28482, P36507, P40763, P42229, P31749, O15111, P29590 | 213 | 58 | 5085 | 3.2928606 | 0.7264061 | 1.22E-01 | 10.832901 |
| T8/NT8 | hsa04370:VEGF signaling pathway | 9 | 1.2658228 | **1.22E-02** | Q92934, P29474, P28482, Q14934, Q16539, P36507, P04792, P47712, P31749 | 213 | 75 | 5085 | 2.8647887 | 0.8022238 | 1.37E-01 | 13.356237 |
| T8/NT8 | hsa04722:Neurotrophin signaling pathway | 12 | 1.6877637 | **1.38E-02** | Q9Y4H2, P05412, P49841, Q92934, P28482, Q16539, Q05655, P36507, P35568, Q99759, P31749, Q99523 | 213 | 124 | 5085 | 2.3103135 | 0.8397247 | 1.42E-01 | 14.952809 |
| T8/NT8 | hsa04664:Fc epsilon RI signaling pathway | 9 | 1.2658228 | **1.53E-02** | Q9UQC2, P30273, P28482, Q16539, Q05655, P36507, P47712, P31749, P07948 | 213 | 78 | 5085 | 2.7546046 | 0.8685133 | 1.44E-01 | 16.429408 |
| T8/NT8 | hsa04510:Focal adhesion | 16 | 2.2503516 | **1.97E-02** | P02751, P18206, Q9Y490, P49841, Q92934, P02452, P21333, P16144, Q13177, P10451, O14974, Q14315, P05412, Q15746, P28482, P31749 | 213 | 201 | 5085 | 1.9003574 | 0.9275308 | 1.71E-01 | 20.719655 |
| T8/NT8 | hsa04920:Adipocytokine signaling pathway | 8 | 1.1251758 | **2.06E-02** | Q9Y4H2, P54646, P35568, Q8N5S9, P40763, P31749, O15111, Q96RR4 | 213 | 67 | 5085 | 2.8505361 | 0.9363084 | 1.68E-01 | 21.619998 |
| T8/NT8 | hsa04620:Toll-like receptor signaling pathway | 10 | 1.4064698 | **2.42E-02** | O43318, Q13546, P05412, P28482, Q16539, Q9NYJ8, P36507, P10451, P31749, O15111 | 213 | 101 | 5085 | 2.3636871 | 0.960387 | 1.83E-01 | 24.844586 |
| T8/NT8 | hsa04012:ErbB signaling pathway | 9 | 1.2658228 | **2.77E-02** | P05412, P49841, Q92934, P28482, Q13177, P36507, P46527, P42229, P31749 | 213 | 87 | 5085 | 2.4696455 | 0.9754317 | 1.96E-01 | 27.954395 |
| T8/NT8 | hsa04330:Notch signaling pathway | 6 | 0.8438819 | **4.45E-02** | Q13547, Q9Y6R0, Q9Y618, Q92769, Q13573, Q8TDB6 | 213 | 47 | 5085 | 3.0476476 | 0.9975498 | 2.84E-01 | 41.245623 |
| T9/NT9 | hsa04530:Tight junction | 13 | 2.5742574 | **1.24E-03** | Q96A65, Q9H4G0, O95049, P35222, P35579, Q9P2M7, P55196, P35580, Q7Z406, P31749, P35749, Q9Y624, Q07157 | 166 | 134 | 5085 | 2.9718126 | 0.1402452 | 1.40E-01 | 1.4159514 |
| T9/NT9 | hsa04510:Focal adhesion | 16 | 3.1683168 | **2.04E-03** | P02751, P18206, Q9Y490, Q92934, P02452, P21333, P16144, P10451, Q15942, P35222, Q14315, P22105, P05412, Q15746, B2ZZ83, P31749 | 166 | 201 | 5085 | 2.4384104 | 0.2204942 | 1.17E-01 | 2.3234027 |
| T9/NT9 | hsa03040:Spliceosome | 12 | 2.3762376 | **2.42E-03** | Q9UKV3, P52272, P26368, O75643, Q13247, P49756, Q13573, Q8NI27, O75533, P61978, Q00839, P38159 | 166 | 126 | 5085 | 2.9173838 | 0.2556182 | 9.37E-02 | 2.7474917 |
| T9/NT9 | hsa05221:Acute myeloid leukemia | 8 | 1.5841584 | **2.49E-03** | Q92934, Q06455, P10398, P36507, P40763, P31749, O15111, P29590 | 166 | 58 | 5085 | 4.2251766 | 0.2620032 | 7.31E-02 | 2.8265259 |
| T9/NT9 | hsa04910:Insulin signaling pathway | 12 | 2.3762376 | **4.14E-03** | Q9Y4H2, Q92934, P54646, Q15642, O95685, P62753, P10398, P36507, P35568, P13861, P46019, P31749 | 166 | 135 | 5085 | 2.7228916 | 0.397191 | 9.63E-02 | 4.6645079 |
| T9/NT9 | hsa04662:B cell receptor signaling pathway | 8 | 1.5841584 | **1.04E-02** | P05412, P29350, O95999, Q9BXL7, Q14934, P36507, P31749, O15111 | 166 | 75 | 5085 | 3.2674699 | 0.719697 | 1.91E-01 | 11.310903 |
| T9/NT9 | hsa04370:VEGF signaling pathway | 8 | 1.5841584 | **1.04E-02** | Q92934, P29474, Q14934, Q16539, P36507, P04792, P47712, P31749 | 166 | 75 | 5085 | 3.2674699 | 0.719697 | 1.91E-01 | 11.310903 |
| T9/NT9 | hsa04920:Adipocytokine signaling pathway | 7 | 1.3861386 | **2.08E-02** | Q9Y4H2, P54646, P35568, Q8N5S9, P40763, P31749, O15111 | 166 | 67 | 5085 | 3.2004136 | 0.9227555 | 3.06E-01 | 21.468611 |
| T9/NT9 | hsa04660:T cell receptor signaling pathway | 9 | 1.7821782 | **2.33E-02** | P05412, P29350, O95999, Q9BXL7, Q14934, Q16539, P36507, P31749, O15111 | 166 | 108 | 5085 | 2.5527108 | 0.9439676 | 3.02E-01 | 23.812309 |
| T9/NT9 | hsa04010:MAPK signaling pathway | 16 | 3.1683168 | **2.57E-02** | P10636, P21333, P15336, Q9Y4G8, P47712, O15111, O95819, Q14315, P05412, P16949, Q14934, Q16539, P36507, B2ZZ83, P04792, P31749 | 166 | 267 | 5085 | 1.8356572 | 0.9582575 | 2.97E-01 | 25.900039 |
| T9/NT9 | hsa04670:Leukocyte transendothelial migration | 9 | 1.7821782 | **3.70E-02** | P55196, P15311, P18206, O60716, P26038, Q96FS4, Q16539, P35222, Q9Y624 | 166 | 118 | 5085 | 2.3363794 | 0.9899851 | 3.69E-01 | 35.23915 |
| T9/NT9 | hsa04520:Adherens junction | 7 | 1.3861386 | **3.80E-02** | P55196, P29350, P18206, Q8WWI1, O60716, P35222, Q07157 | 166 | 77 | 5085 | 2.7847755 | 0.9910909 | 3.49E-01 | 35.950324 |
| T9/NT9 | hsa04810:Regulation of actin cytoskeleton | 13 | 2.5742574 | **4.56E-02** | P02751, Q9NYB9, P18206, P26038, P16144, P10398, P35579, P35580, Q15746, P15311, Q7Z406, P36507, Q13576 | 166 | 215 | 5085 | 1.8521995 | 0.9966421 | 3.78E-01 | 41.584981 |
| T10/NT10 | hsa04530:Tight junction | 18 | 2.6509573 | **5.76E-05** | Q9H4G0, O95049, P35221, P11171, P35222, P35579, Q13813, P55196, Q9P2M7, P35580, Q5JTD0, Q7Z406, Q8NI35, Q9Y624, P31749, P35749, Q07157, Q14247 | 222 | 134 | 5085 | 3.0768455 | 0.0072945 | 7.29E-03 | 0.0668322 |
| T10/NT10 | hsa04510:Focal adhesion | 21 | 3.0927835 | **3.95E-04** | P18206, Q9Y490, P49841, Q92934, P02452, P16144, P21333, Q13177, Q9Y4G6, P10451, Q15942, P35222, O14974, P04049, P22105, P05412, Q13905, Q05397, P49023, P28482, P31749 | 222 | 201 | 5085 | 2.3931021 | 0.0489262 | 1.25E-02 | 0.4570234 |
| T10/NT10 | hsa04370:VEGF signaling pathway | 12 | 1.7673049 | **3.42E-04** | P04049, Q92934, Q05397, P49023, P29474, P28482, Q14934, Q08209, Q16539, P36507, P04792, P31749 | 222 | 75 | 5085 | 3.6648649 | 0.0424668 | 1.44E-02 | 0.3954783 |
| T10/NT10 | hsa04012:ErbB signaling pathway | 13 | 1.9145803 | **3.32E-04** | Q92934, P49841, Q13177, P10398, P42229, P04049, P05412, Q05397, P28482, P16333, P36507, P31749, P46527 | 222 | 87 | 5085 | 3.4226468 | 0.0412915 | 2.09E-02 | 0.3843201 |
| T10/NT10 | hsa03040:Spliceosome | 15 | 2.2091311 | **1.03E-03** | P52272, O75643, Q13573, O60231, Q13247, P49756, Q07955, Q9UKV3, Q08170, Q9Y5S9, P08621, P61978, P09651, Q15365, P38159 | 222 | 126 | 5085 | 2.726834 | 0.1224251 | 2.15E-02 | 1.1854332 |
| T10/NT10 | hsa04810:Regulation of actin cytoskeleton | 21 | 3.0927835 | **9.40E-04** | Q9NYB9, P18206, P26038, P16144, P10398, Q13177, Q9NZN5, O14974, P35579, Q14155, P35580, Q9UBI6, P04049, Q9Y2I7, Q05397, Q15052, Q7Z406, P49023, P28482, P36507, Q13576 | 222 | 215 | 5085 | 2.2372722 | 0.1126095 | 2.36E-02 | 1.0850175 |
| T10/NT10 | hsa05213:Endometrial cancer | 9 | 1.3254786 | **1.61E-03** | P04049, P49841, Q92934, P28482, P35221, P10398, P36507, P35222, P31749 | 222 | 52 | 5085 | 3.9643971 | 0.1853304 | 2.89E-02 | 1.8543085 |
| T10/NT10 | hsa04660:T cell receptor signaling pathway | 13 | 1.9145803 | **2.33E-03** | O95999, P49841, Q08209, Q13177, P04049, P05412, Q14934, P28482, P16333, Q16539, P36507, P31749, Q12959 | 222 | 108 | 5085 | 2.7571321 | 0.2565939 | 3.64E-02 | 2.671292 |
| T10/NT10 | hsa05221:Acute myeloid leukemia | 9 | 1.3254786 | **3.28E-03** | P04049, Q92934, P28482, P10398, P36507, P40763, P42229, P31749, P29590 | 222 | 58 | 5085 | 3.554287 | 0.3414775 | 4.09E-02 | 3.742917 |
| T10/NT10 | hsa04270:Vascular smooth muscle contraction | 13 | 1.9145803 | **3.17E-03** | Q14573, P10398, Q9NZN5, O14974, O60237, Q96A00, Q9Y6F6, P04049, Q05682, P28482, P36507, O15085, P35749 | 222 | 112 | 5085 | 2.6586631 | 0.3318033 | 4.38E-02 | 3.6146426 |
| T10/NT10 | hsa04662:B cell receptor signaling pathway | 10 | 1.4727541 | **4.84E-03** | P04049, P05412, O95999, P49841, P28482, Q14934, Q08209, P36507, P31749, P07948 | 222 | 75 | 5085 | 3.0540541 | 0.4603011 | 5.45E-02 | 5.4761706 |
| T10/NT10 | hsa05220:Chronic myeloid leukemia | 9 | 1.3254786 | **1.54E-02** | Q13547, P04049, Q92934, P28482, P10398, P36507, P46527, P42229, P31749 | 222 | 75 | 5085 | 2.7486486 | 0.8605715 | 1.23E-01 | 16.465458 |
| T10/NT10 | hsa05215:Prostate cancer | 10 | 1.4727541 | **1.45E-02** | P04049, P14625, P49841, Q92934, P28482, P10398, P36507, P35222, P46527, P31749 | 222 | 89 | 5085 | 2.5736411 | 0.8440867 | 1.24E-01 | 15.608679 |
| T10/NT10 | hsa00010:Glycolysis / Gluconeogenesis | 8 | 1.1782032 | **1.45E-02** | P04406, P60174, P36871, P18669, Q96G03, P04075, P08559, P17858 | 222 | 60 | 5085 | 3.0540541 | 0.8430846 | 1.33E-01 | 15.559291 |
| T10/NT10 | hsa04722:Neurotrophin signaling pathway | 12 | 1.7673049 | **1.83E-02** | P04049, P05412, Q13905, Q9Y5V3, P49841, Q92934, P28482, Q9ULH0, Q16539, P36507, P35568, P31749 | 222 | 124 | 5085 | 2.2166521 | 0.904157 | 1.36E-01 | 19.276359 |
| T10/NT10 | hsa04910:Insulin signaling pathway | 13 | 1.9145803 | **1.38E-02** | Q92934, P49841, Q15642, O95685, P10398, P35568, P13861, P04049, Q13905, P28482, P36507, P31749, P46019 | 222 | 135 | 5085 | 2.2057057 | 0.8289625 | 1.37E-01 | 14.892193 |
| T10/NT10 | hsa04666:Fc gamma R-mediated phagocytosis | 10 | 1.4727541 | **2.15E-02** | Q92608, P04049, Q9Y2I7, Q9ULH1, P28482, P29966, O43150, P49006, P31749, P07948 | 222 | 95 | 5085 | 2.4110953 | 0.936581 | 1.42E-01 | 22.263646 |
| T10/NT10 | hsa00030:Pentose phosphate pathway | 5 | 0.736377 | **2.14E-02** | P37837, P36871, Q96G03, P04075, P17858 | 222 | 25 | 5085 | 4.5810811 | 0.9359705 | 1.49E-01 | 22.195613 |
| T10/NT10 | hsa04144:Endocytosis | 15 | 2.2091311 | **2.83E-02** | Q8NDX1, Q9ULH1, Q15276, Q8N6H7, O43150, Q9NP61, Q9BXF6, Q99961, Q9Y2I7, Q96PU5, Q9UQN3, Q96B97, Q15075, Q00610, Q7L804 | 222 | 184 | 5085 | 1.8672885 | 0.9740198 | 1.67E-01 | 28.347314 |
| T10/NT10 | hsa04720:Long-term potentiation | 8 | 1.1782032 | **2.72E-02** | P04049, Q14573, Q13522, P28482, Q08209, P10398, P36507, O14974 | 222 | 68 | 5085 | 2.6947536 | 0.9697753 | 1.68E-01 | 27.350326 |
| T10/NT10 | hsa04670:Leukocyte transendothelial migration | 11 | 1.6200295 | **3.16E-02** | P55196, P18206, Q05397, P49023, O60716, P26038, Q96FS4, P35221, Q16539, P35222, Q9Y624 | 222 | 118 | 5085 | 2.1352497 | 0.9831067 | 1.69E-01 | 31.108923 |
| T10/NT10 | hsa05211:Renal cell carcinoma | 8 | 1.1782032 | **3.13E-02** | P04049, P05412, Q13905, P28482, P10398, Q13177, P36507, P31749 | 222 | 70 | 5085 | 2.6177606 | 0.982283 | 1.75E-01 | 30.808793 |
| T10/NT10 | hsa05216:Thyroid cancer | 5 | 0.736377 | **3.51E-02** | P12270, P28482, P36507, P35222, Q16204 | 222 | 29 | 5085 | 3.9492078 | 0.9893578 | 1.79E-01 | 33.95539 |
| T10/NT10 | hsa04010:MAPK signaling pathway | 19 | 2.7982327 | **4.10E-02** | P10636, Q08209, P21333, P15336, Q9Y4G8, Q13177, O95819, Q9UBI6, P04049, P05412, P16949, P28482, Q14934, P11831, Q16539, P36507, Q9NYJ8, P04792, P31749 | 222 | 267 | 5085 | 1.6299727 | 0.9951076 | 1.92E-01 | 38.479787 |
| T10/NT10 | hsa00520:Amino sugar and nucleotide sugar metabolism | 6 | 0.8836524 | **4.06E-02** | Q06210, P36871, Q96G03, Q9UJ70, O60701, O95394 | 222 | 44 | 5085 | 3.1234644 | 0.9947976 | 1.97E-01 | 38.133693 |
| T11/NT11 | hsa04530:Tight junction | 23 | 3.1420765 | **6.44E-08** | O14493, Q9H4G0, P16989, Q3MNF1, Q9NYL2, O95049, Q9UDY2, P35221, Q05655, P35579, Q13813, O43491, P55196, Q9P2M7, P35580, Q16625, Q5JTD0, Q7Z406, Q8NI35, O43707, Q9Y624, P78369, P35749, Q07157 | 226 | 134 | 5085 | 3.8619403 | 8.56E-06 | 8.56E-06 | 7.53E-05 |
| T11/NT11 | hsa04270:Vascular smooth muscle contraction | 16 | 2.1857923 | **1.04E-04** | Q14573, P63092, Q3MNF1, Q13464, P10398, Q05655, O60237, Q9NZN5, O14974, Q96A00, Q9Y6F6, P04049, Q05682, P28482, P36507, O15085, P35749 | 226 | 112 | 5085 | 3.2142857 | 0.0137585 | 6.90E-03 | 0.1217248 |
| T11/NT11 | hsa04810:Regulation of actin cytoskeleton | 23 | 3.1420765 | **1.74E-04** | P08648, P18206, P26038, Q13464, Q9UQB8, P10398, Q9NZN5, O14974, P35579, Q14155, P35580, P04049, Q15052, Q7Z406, P49023, P25054, P26010, P28482, Q76I76, O43707, Q9NRY4, P36507, Q13576 | 226 | 215 | 5085 | 2.4069767 | 0.0229065 | 7.69E-03 | 0.2035185 |
| T11/NT11 | hsa04670:Leukocyte transendothelial migration | 15 | 2.0491803 | **6.35E-04** | O14493, P18206, P26038, Q96FS4, Q13464, P35221, P55196, Q16625, P49023, Q15080, O60716, Q9NRY4, O43707, Q9Y624, P78369 | 226 | 118 | 5085 | 2.8601695 | 0.0809759 | 2.09E-02 | 0.7396365 |
| T11/NT11 | hsa03040:Spliceosome | 15 | 2.0491803 | **1.22E-03** | P52272, P51991, Q13573, Q13247, Q07955, Q9UKV3, Q16629, Q9Y5S9, P08621, Q5VTL8, Q13242, P61978, P09651, Q15365, P38159 | 226 | 126 | 5085 | 2.6785714 | 0.1502805 | 3.20E-02 | 1.4214971 |
| T11/NT11 | hsa04510:Focal adhesion | 19 | 2.5956284 | **3.15E-03** | P08648, P18206, Q9Y490, Q92934, Q13464, P21333, P10451, Q15942, O14974, Q03135, Q14315, P04049, P05412, P49023, Q9NVD7, P26010, P28482, Q9NRY4, O43707 | 226 | 201 | 5085 | 2.1268657 | 0.3425831 | 6.75E-02 | 3.6203464 |
| T11/NT11 | hsa04520:Adherens junction | 10 | 1.3661202 | **6.48E-03** | P55196, O43318, P18206, Q8WWI1, O60716, P28482, Q9UQB8, P35221, O43707, Q07157 | 226 | 77 | 5085 | 2.9220779 | 0.5788665 | 1.02E-01 | 7.3211535 |
| T11/NT11 | hsa05213:Endometrial cancer | 8 | 1.0928962 | **7.39E-03** | P04049, Q92934, P25054, P28482, P35221, P10398, P36507, O43524 | 226 | 52 | 5085 | 3.4615385 | 0.6273717 | 1.04E-01 | 8.3128594 |
| T11/NT11 | hsa04722:Neurotrophin signaling pathway | 13 | 1.7759563 | **8.30E-03** | Q13233, Q92934, Q9ULH0, Q05655, P35568, O43524, Q99523, Q9Y4H2, P04049, Q13480, P05412, P28482, P36507 | 226 | 124 | 5085 | 2.358871 | 0.6699201 | 1.05E-01 | 9.2850054 |
| T11/NT11 | hsa05412:Arrhythmogenic right ventricular cardiomyopathy (ARVC) | 10 | 1.3661202 | **5.95E-03** | P02545, P08648, P15924, Q14126, P26010, P35221, O43707, Q99959, P16615, P11532 | 226 | 76 | 5085 | 2.9605263 | 0.5476174 | 1.07E-01 | 6.7360995 |
| T11/NT11 | hsa03010:Ribosome | 10 | 1.3661202 | **1.41E-02** | P15880, P55795, P05387, P05386, P62888, P61247, P62753, P42677, P23396, P26373 | 226 | 87 | 5085 | 2.5862069 | 0.8483653 | 1.45E-01 | 15.281282 |
| T11/NT11 | hsa05221:Acute myeloid leukemia | 8 | 1.0928962 | **1.33E-02** | P04049, Q7Z4J5, Q92934, P28482, P10398, P36507, P42229, P29590 | 226 | 58 | 5085 | 3.1034483 | 0.8313104 | 1.49E-01 | 14.483689 |
| T11/NT11 | hsa04910:Insulin signaling pathway | 13 | 1.7759563 | **1.57E-02** | Q13131, Q93100, Q92934, O95685, P62753, P10398, P35568, P13861, Q9Y4H2, P04049, P28482, Q16822, P36507 | 226 | 135 | 5085 | 2.1666667 | 0.8786906 | 1.50E-01 | 16.926986 |
| T11/NT11 | hsa04120:Ubiquitin mediated proteolysis | 13 | 1.7759563 | **1.75E-02** | O15344, Q9C0C9, Q13233, Q15751, Q7Z6Z7, Q9Y385, Q14669, Q9UJX2, O00308, Q92466, O95071, Q8NHZ8, P29590 | 226 | 137 | 5085 | 2.1350365 | 0.9045369 | 1.54E-01 | 18.658616 |
| T11/NT11 | hsa04912:GnRH signaling pathway | 10 | 1.3661202 | **2.85E-02** | P04049, P05412, Q14573, P63092, Q13233, Q9Y2U5, P52564, P28482, Q05655, P36507 | 226 | 98 | 5085 | 2.2959184 | 0.9785959 | 2.26E-01 | 28.677823 |
| T11/NT11 | hsa04370:VEGF signaling pathway | 8 | 1.0928962 | **4.69E-02** | P04049, Q92934, P49023, P29474, P28482, Q14934, P36507, P04792 | 226 | 75 | 5085 | 2.4 | 0.9983097 | 3.29E-01 | 42.945012 |
| T11/NT11 | hsa05220:Chronic myeloid leukemia | 8 | 1.0928962 | **4.69E-02** | P04049, Q9UQC2, P11274, Q92934, P28482, P10398, P36507, P42229 | 226 | 75 | 5085 | 2.4 | 0.9983097 | 3.29E-01 | 42.945012 |
| T12/NT12 | hsa04530:Tight junction | 4 | 5.5555556 | **1.68E-02** | O14493, Q9H4G0, O95049, P78369 | 22 | 134 | 5085 | 6.8995929 | 0.5094701 | 5.09E-01 | 14.5256 |
| T12/NT12 | hsa05412:Arrhythmogenic right ventricular cardiomyopathy (ARVC) | 3 | 4.1666667 | **3.85E-02** | P15924, Q99959, P11532 | 22 | 76 | 5085 | 9.1238038 | 0.8080886 | 5.62E-01 | 30.493339 |
| T12/NT12 | hsa04510:Focal adhesion | 4 | 5.5555556 | **4.79E-02** | Q9Y490, Q9NVD7, Q14185, Q07889 | 22 | 201 | 5085 | 4.5997286 | 0.8727504 | 4.97E-01 | 36.510042 |
| T13/NT13 | hsa04530:Tight junction | 20 | 3.8314176 | **6.65E-09** | O14493, Q9H4G0, P12931, Q3MNF1, Q9NYL2, Q6P1M3, O95049, Q9UDY2, P35221, P35222, P35579, Q13813, P55196, Q9P2M7, Q7Z406, Q8NI35, P31749, P78369, P35749, Q07157, Q14247 | 149 | 134 | 5085 | 5.0936592 | 6.91E-07 | 6.91E-07 | 7.43E-06 |
| T13/NT13 | hsa03040:Spliceosome | 16 | 3.0651341 | **2.86E-06** | Q8WWY3, P51991, O60231, Q9NW64, Q13247, Q07955, P08107, Q9UKV3, P62995, P08621, Q5VTL8, Q13242, P61978, P09651, Q15365, P38159 | 149 | 126 | 5085 | 4.3336529 | 2.98E-04 | 1.49E-04 | 0.0032018 |
| T13/NT13 | hsa04510:Focal adhesion | 17 | 3.256705 | **2.11E-04** | P18206, P12931, Q9Y490, Q92934, P02452, P21333, P35222, O14974, Q14315, P04049, P05412, Q15746, P49023, Q9NVD7, Q14185, P46108, P31749 | 149 | 201 | 5085 | 2.8864069 | 0.0217139 | 7.29E-03 | 0.2357442 |
| T13/NT13 | hsa04520:Adherens junction | 10 | 1.9157088 | **3.50E-04** | P55196, P18206, P12931, Q8WWI1, O60716, Q9UQB8, P35221, P35222, P18031, Q07157 | 149 | 77 | 5085 | 4.432145 | 0.0357818 | 9.07E-03 | 0.3909831 |
| T13/NT13 | hsa04270:Vascular smooth muscle contraction | 11 | 2.1072797 | **1.43E-03** | P04049, Q14573, P63092, Q15746, Q05682, Q3MNF1, P10398, Q14643, O60237, O14974, P35749, Q9Y6F6 | 149 | 112 | 5085 | 3.3518097 | 0.1385427 | 2.94E-02 | 1.5905425 |
| T13/NT13 | hsa05220:Chronic myeloid leukemia | 8 | 1.532567 | **5.82E-03** | Q13547, P04049, Q92934, Q92769, P10398, P46108, P42229, P31749 | 149 | 75 | 5085 | 3.6402685 | 0.4551154 | 9.62E-02 | 6.3194314 |
| T13/NT13 | hsa05412:Arrhythmogenic right ventricular cardiomyopathy (ARVC) | 8 | 1.532567 | **6.26E-03** | P02545, P15924, Q14126, P35221, P17661, Q99959, P35222, P11532 | 149 | 76 | 5085 | 3.5923702 | 0.4795188 | 8.91E-02 | 6.7797895 |
| T13/NT13 | hsa04012:ErbB signaling pathway | 8 | 1.532567 | **1.28E-02** | P04049, P05412, P12931, Q92934, P10398, P46108, P42229, P31749 | 149 | 87 | 5085 | 3.1381625 | 0.7392836 | 1.55E-01 | 13.457195 |
| T13/NT13 | hsa04540:Gap junction | 8 | 1.532567 | **1.44E-02** | P04049, Q14573, P63092, P12931, Q9Y2U5, Q14643, Q07157, P48730 | 149 | 89 | 5085 | 3.067642 | 0.7795733 | 1.55E-01 | 15.005095 |
| T13/NT13 | hsa04910:Insulin signaling pathway | 10 | 1.9157088 | **1.63E-02** | Q13131, P04049, Q92934, Q8N122, O95685, P10398, P46108, P13861, P18031, P31749 | 149 | 135 | 5085 | 2.5279642 | 0.818809 | 1.57E-01 | 16.777525 |
| T13/NT13 | hsa05213:Endometrial cancer | 6 | 1.1494253 | **1.69E-02** | P04049, Q92934, P35221, P10398, P35222, P31749 | 149 | 52 | 5085 | 3.9377904 | 0.8300959 | 1.49E-01 | 17.351015 |
| T13/NT13 | hsa04670:Leukocyte transendothelial migration | 9 | 1.7241379 | **2.10E-02** | P55196, O14493, P18206, Q15080, P49023, O60716, P35221, P35222, P78369 | 149 | 118 | 5085 | 2.6029462 | 0.8900443 | 1.68E-01 | 21.128666 |
| T13/NT13 | hsa04370:VEGF signaling pathway | 7 | 1.3409962 | **2.13E-02** | P04049, P12931, Q92934, P49023, P29474, P04792, P31749 | 149 | 75 | 5085 | 3.1852349 | 0.893129 | 1.58E-01 | 21.369585 |
| T13/NT13 | hsa05221:Acute myeloid leukemia | 6 | 1.1494253 | **2.60E-02** | P04049, Q92934, P10398, P42229, P31749, P29590 | 149 | 58 | 5085 | 3.5304328 | 0.9350912 | 1.77E-01 | 25.47394 |
| T13/NT13 | hsa04720:Long-term potentiation | 6 | 1.1494253 | **4.70E-02** | P04049, Q14573, Q13522, P10398, Q14643, O14974 | 149 | 68 | 5085 | 3.0112515 | 0.9932899 | 2.84E-01 | 41.608884 |
| T13/NT13 | hsa04810:Regulation of actin cytoskeleton | 12 | 2.2988506 | **4.77E-02** | P04049, P18206, Q15746, Q7Z406, P49023, Q14185, Q9UQB8, P19634, P10398, P46108, O14974, P35579 | 149 | 215 | 5085 | 1.9047916 | 0.9938063 | 2.72E-01 | 42.109451 |
| T14/NT14 | hsa04530:Tight junction | 20 | 3.2894737 | **3.65E-07** | O14493, Q9H4G0, Q3MNF1, P16989, Q6P1M3, O95049, Q9UDY2, P35221, P35222, P35579, P55196, Q9P2M7, P35580, Q16625, Q7Z406, Q8NI35, Q9Y624, P78369, P35749, Q07157, Q14247 | 190 | 134 | 5085 | 3.9945012 | 4.79E-05 | 4.79E-05 | 4.26E-04 |
| T14/NT14 | hsa03040:Spliceosome | 16 | 2.6315789 | **5.58E-05** | P52272, Q13573, O60231, Q13247, P49756, Q07955, Q9UKV3, Q16629, P62995, Q5VTL8, Q13242, P61978, Q00839, P09651, Q15365, P38159 | 190 | 126 | 5085 | 3.3984962 | 0.0072869 | 3.65E-03 | 0.0650832 |
| T14/NT14 | hsa04270:Vascular smooth muscle contraction | 13 | 2.1381579 | **8.32E-04** | P63092, Q3MNF1, Q13464, P10398, Q9NZN5, O60237, P47712, Q96A00, Q9Y6F6, P04049, Q15746, P36507, O15085, P35749 | 190 | 112 | 5085 | 3.106438 | 0.1032587 | 3.57E-02 | 0.965499 |
| T14/NT14 | hsa04810:Regulation of actin cytoskeleton | 19 | 3.125 | **9.41E-04** | P18206, O60610, Q13464, P19634, P10398, Q9NZN5, P35579, Q14155, P35580, P04049, Q15746, Q15052, Q7Z406, P25054, P26010, Q9Y2X7, Q14185, P36507, Q13576 | 190 | 215 | 5085 | 2.3651163 | 0.1160829 | 3.04E-02 | 1.0924018 |
| T14/NT14 | hsa04910:Insulin signaling pathway | 13 | 2.1381579 | **4.13E-03** | Q13131, Q92934, Q308M2, Q14432, O95685, P49815, P62753, P10398, P13861, Q9Y4H2, P04049, Q16822, P36507 | 190 | 135 | 5085 | 2.577193 | 0.4184421 | 1.03E-01 | 4.7106193 |
| T14/NT14 | hsa03010:Ribosome | 10 | 1.6447368 | **4.69E-03** | P15880, P55795, P05387, P05386, P62888, P61247, P62753, P42677, P23396, P26373 | 190 | 87 | 5085 | 3.076225 | 0.4596029 | 9.75E-02 | 5.3312569 |
| T14/NT14 | hsa05130:Pathogenic Escherichia coli infection | 8 | 1.3157895 | **4.80E-03** | O43639, Q92974, Q16625, Q13464, P05783, P35222, P19338, Q14247 | 190 | 57 | 5085 | 3.7562327 | 0.4676489 | 8.61E-02 | 5.4575902 |
| T14/NT14 | hsa05412:Arrhythmogenic right ventricular cardiomyopathy (ARVC) | 9 | 1.4802632 | **6.80E-03** | P02545, P15924, Q14126, P26010, P35221, P17661, Q99959, P35222, P11532 | 190 | 76 | 5085 | 3.1693213 | 0.5908833 | 1.06E-01 | 7.6477751 |
| T14/NT14 | hsa04520:Adherens junction | 9 | 1.4802632 | **7.35E-03** | P55196, P08069, O43318, P18206, Q8WWI1, O60716, P35221, P35222, Q07157 | 190 | 77 | 5085 | 3.1281613 | 0.6196813 | 1.02E-01 | 8.2458909 |
| T14/NT14 | hsa04510:Focal adhesion | 16 | 2.6315789 | **7.38E-03** | P18206, O60610, Q92934, Q308M2, Q13464, P21333, P10451, P35222, Q14315, P04049, P08069, P05412, Q15746, Q9NVD7, P26010, Q14185 | 190 | 201 | 5085 | 2.1304006 | 0.6210144 | 9.25E-02 | 8.2745655 |
| T14/NT14 | hsa05213:Endometrial cancer | 7 | 1.1513158 | **1.20E-02** | P04049, Q92934, P25054, P35221, P10398, P36507, P35222 | 190 | 52 | 5085 | 3.6027328 | 0.7931399 | 1.33E-01 | 13.087384 |
| T14/NT14 | hsa04670:Leukocyte transendothelial migration | 10 | 1.6447368 | **3.06E-02** | P55196, O14493, P18206, Q16625, O60716, Q13464, P35221, P35222, Q9Y624, P78369 | 190 | 118 | 5085 | 2.2680642 | 0.983051 | 2.88E-01 | 30.439663 |
| T14/NT14 | hsa05210:Colorectal cancer | 8 | 1.3157895 | **3.57E-02** | P08069, P04049, P05412, Q92934, Q308M2, P25054, P10398, P35222 | 190 | 84 | 5085 | 2.5488722 | 0.9914331 | 3.07E-01 | 34.538812 |
| T14/NT14 | hsa04012:ErbB signaling pathway | 8 | 1.3157895 | **4.20E-02** | P04049, P05412, O43639, Q92934, Q308M2, P10398, P36507, P42229 | 190 | 87 | 5085 | 2.46098 | 0.9963749 | 3.31E-01 | 39.363299 |

Table S8.

| CASE # | Uniprot | Protein | Peptide | T/NT | Global | Phos Site 1_Function | Phos Site 2_Function | Drug |
| --- | --- | --- | --- | --- | --- | --- | --- | --- |
| 1 | P47712 | Cytosolic phospholipase A2 | qNPSRcsVSLSNVEAR | 1.63 | S727 | enzymatic activity, induced |  |  |
| 1 | P49840 | Glycogen synthase kinase-3 alpha | qLVRGEPNVSyIcSR | 1.14 | Y279 | enzymatic activity, induced |  |  |
| 1 | P08559 | Pyruvate dehydrogenase E1 component subunit alpha, somatic form, mitochondrial | YGMGTsVER | -1.53 | S232 | enzymatic activity, inhibited |  |  |
| 1 | P08559 | Pyruvate dehydrogenase E1 component subunit alpha, somatic form, mitochondrial | YGmGTsVER | -1.67 | S232 | enzymatic activity, inhibited |  |  |
| 1 | P08559 | Pyruvate dehydrogenase E1 component subunit alpha, somatic form, mitochondrial | YHGHsmSDPGVsYR | -1.10 | S293;S300 | enzymatic activity, inhibited | enzymatic activity, inhibited |  |
| 1 | P31749 | RAC-alpha serine/threonine-protein kinase | SGsPSDNSGAEEMEVSLAkPk | -1.28 | S124 | enzymatic activity, induced |  | GSK2141795 |
| 1 | P31749 | RAC-alpha serine/threonine-protein kinase | SGsPSDNSGAEEmEVSLAkPk | -1.16 | S124 | enzymatic activity, induced |  | GSK2141796 |
| 1 | P31749 | RAC-alpha serine/threonine-protein kinase | SGsPSDNsGAEEMEVSLAkPk | -1.04 | S124;S129 | enzymatic activity, induced | enzymatic activity, induced | GSK2141797 |
| 1 | P06241 | Tyrosine-protein kinase Fyn | lTEERDGsLNQSSGYR | 1.15 | S21 | enzymatic activity, induced |  | Dasatinib |
|  |  |  |  |  |  |  |  |  |
| 4 | P28482 | Mitogen-activated protein kinase 1 | vADPDHDHTGFLtEyVATR | -1.93 | T185;Y187 | enzymatic activity, induced | enzymatic activity, induced | AEZS-131 |
| 4 | P31749 | RAC-alpha serine/threonine-protein kinase | SGsPSDNSGAEEMEVSLAkPk | 1.69 | S124 | enzymatic activity, induced |  | GSK2141795 |
| 4 | P31749 | RAC-alpha serine/threonine-protein kinase | SGsPSDNSGAEEmEVSLAkPk | 1.59 | S124 | enzymatic activity, induced |  | GSK2141796 |
| 4 | P31749 | RAC-alpha serine/threonine-protein kinase | SGsPSDNsGAEEMEVSLAkPk | 1.31 | S124;S129 | enzymatic activity, induced | enzymatic activity, induced | GSK2141797 |
|  |  |  |  |  |  |  |  |  |
| 5 | P13569 | Cystic fibrosis transmembrane conductance regulator | rLsLVPDSEQGEAILPR | -1.11 | S737 | enzymatic activity, inhibited |  |  |
| 5 | Q06210 | Glucosamine--fructose-6-phosphate aminotransferase [isomerizing] 1 | gScNLSRVDsTTcLFPVEEk | -1.02 | S261 | enzymatic activity, induced |  |  |
| 5 | Q06210 | Glucosamine--fructose-6-phosphate aminotransferase [isomerizing] 1 | vDsTTcLFPVEEk | -1.25 | S261 | enzymatic activity, induced |  |  |
| 5 | P28482 | Mitogen-activated protein kinase 1 | vADPDHDHTGFLtEyVATR | 1.05 | T185;Y187 | enzymatic activity, induced | enzymatic activity, induced | AEZS-131 |
| 5 | P08559 | Pyruvate dehydrogenase E1 component subunit alpha, somatic form, mitochondrial | YGMGTsVER | -1.41 | S232 | enzymatic activity, inhibited |  |  |
| 5 | P08559 | Pyruvate dehydrogenase E1 component subunit alpha, somatic form, mitochondrial | YGmGTsVER | -1.82 | S232 | enzymatic activity, inhibited |  |  |
| 5 | P08559 | Pyruvate dehydrogenase E1 component subunit alpha, somatic form, mitochondrial | YHGHsmSDPGVsYR | -1.53 | S293;S300 | enzymatic activity, inhibited | enzymatic activity, inhibited |  |
|  |  |  |  |  |  |  |  |  |
| 6 | Q06210 | Glucosamine--fructose-6-phosphate aminotransferase [isomerizing] 1 | gScNLSRVDsTTcLFPVEEk | -1.33 | S261 | enzymatic activity, induced |  |  |
| 6 | Q06210 | Glucosamine--fructose-6-phosphate aminotransferase [isomerizing] 1 | vDsTTcLFPVEEk | -1.14 | S261 | enzymatic activity, induced |  |  |
| 6 | P49840 | Glycogen synthase kinase-3 alpha | gEPNVSyIcSR | 1.03 | Y279 | enzymatic activity, induced |  |  |
| 6 | P31749 | RAC-alpha serine/threonine-protein kinase | SGsPSDNsGAEEMEVSLAkPk | -1.10 | S124;S129 | enzymatic activity, induced | enzymatic activity, induced | GSK2141795 |
|  |  |  |  |  |  |  |  |  |
| 7 | Q06210 | Glucosamine--fructose-6-phosphate aminotransferase [isomerizing] 1 | gScNLSRVDsTTcLFPVEEk | 1.11 | S261 | enzymatic activity, induced |  |  |
| 7 | P49841 | Glycogen synthase kinase-3 beta | TTsFAESckPVQQPSAFGSmk | -1.11 | S9 | enzymatic activity, inhibited |  |  |
| 7 | P31749 | RAC-alpha serine/threonine-protein kinase | SGsPSDNsGAEEMEVSLAkPk | 1.12 | S124;S129 | enzymatic activity, induced | enzymatic activity, induced | GSK2141795 |
| 7 | P06241 | Tyrosine-protein kinase Fyn | dGsLNQSSGYR | -1.14 | S21 | enzymatic activity, induced |  | Dasatinib |
|  |  |  |  |  |  |  |  |  |
| 8 | P13569 | Cystic fibrosis transmembrane conductance regulator | rLsLVPDSEQGEAILPR | -4.50 | S737 | enzymatic activity, inhibited |  |  |
| 8 | Q06210 | Glucosamine--fructose-6-phosphate aminotransferase [isomerizing] 1 | vDsTTcLFPVEEk | -1.13 | S261 | enzymatic activity, induced |  |  |
| 8 | P49841 | Glycogen synthase kinase-3 beta | TTsFAESckPVQQPSAFGSMk | 2.05 | S9 | enzymatic activity, inhibited |  |  |
| 8 | P49841 | Glycogen synthase kinase-3 beta | TTsFAESckPVQQPSAFGSmk | 1.23 | S9 | enzymatic activity, inhibited |  |  |
| 8 | P49841 | Glycogen synthase kinase-3 beta | gEPNVSyIcSR | 2.32 | Y216 | enzymatic activity, induced |  |  |
| 8 | P49841 | Glycogen synthase kinase-3 beta | qLVRGEPNVSyIcSR | 1.13 | Y216 | enzymatic activity, induced |  |  |
| 8 | Q13547 | Histone deacetylase 1 | iAcEEEFsDsEEEGEGGRk | -1.04 | S421;S423 | enzymatic activity, induced | enzymatic activity, induced | Vorinostat |
| 8 | Q92769 | Histone deacetylase 2 | iAcDEEFsDSEDEGEGGRR | 1.43 | S422 | enzymatic activity, inhibited |  | Vorinostat |
| 8 | P28482 | Mitogen-activated protein kinase 1 | vADPDHDHTGFLtEyVATR | 1.44 | T185;Y187 | enzymatic activity, induced | enzymatic activity, induced | AEZS-131 |
| 8 | Q16539 | Mitogen-activated protein kinase 14 | hTDDEMtGyVATR | 1.33 | T180;Y182 | enzymatic activity, induced | enzymatic activity, induced |  |
| 8 | Q16539 | Mitogen-activated protein kinase 14 | hTDDEMTGyVATR | -1.34 | Y182 | enzymatic activity, induced |  |  |
| 8 | P29474 | Nitric oxide synthase, endothelial | iRtQsFSLQER | -1.23 | T1175;S1177 |  | enzymatic activity, induced |  |
| 8 | P29474 | Nitric oxide synthase, endothelial | iRtQSFsLQER | -1.77 | T1175;S1179 |  | enzymatic activity, induced |  |
| 8 | P08559 | Pyruvate dehydrogenase E1 component subunit alpha, somatic form, mitochondrial | YGmGTsVER | -2.68 | S232 | enzymatic activity, inhibited |  |  |
| 8 | P08559 | Pyruvate dehydrogenase E1 component subunit alpha, somatic form, mitochondrial | YHGHsmSDPGVsYR | -1.44 | S293;S300 | enzymatic activity, inhibited | enzymatic activity, inhibited |  |
| 8 | P08559 | Pyruvate dehydrogenase E1 component subunit alpha, somatic form, mitochondrial | YHGHsMSDPGVsYR | -1.27 | S293;S300 | enzymatic activity, inhibited | enzymatic activity, inhibited |  |
| 8 | P31749 | RAC-alpha serine/threonine-protein kinase | SGsPSDNSGAEEMEVSLAkPk | -1.57 | S124 | enzymatic activity, induced |  | GSK2141795 |
| 8 | P31749 | RAC-alpha serine/threonine-protein kinase | SGsPSDNsGAEEMEVSLAkPk | -2.86 | S124;S129 | enzymatic activity, induced | enzymatic activity, induced | GSK2141795 |
|  |  |  |  |  |  |  |  |  |
| 9 | P13569 | Cystic fibrosis transmembrane conductance regulator | rLsLVPDSEQGEAILPR | -2.35 | S737 | enzymatic activity, inhibited |  |  |
| 9 | Q06210 | Glucosamine--fructose-6-phosphate aminotransferase [isomerizing] 1 | gScNLSRVDsTTcLFPVEEk | -1.28 | S261 | enzymatic activity, induced |  |  |
| 9 | Q06210 | Glucosamine--fructose-6-phosphate aminotransferase [isomerizing] 1 | vDsTTcLFPVEEk | -2.06 | S261 | enzymatic activity, induced |  |  |
| 9 | Q16539 | Mitogen-activated protein kinase 14 | hTDDEMtGyVATR | 1.39 | T180;Y182 | enzymatic activity, induced | enzymatic activity, induced |  |
| 9 | P29474 | Nitric oxide synthase, endothelial | iRtQSFsLQER | -1.22 | T1175;S1179 |  | enzymatic activity, induced |  |
| 9 | P08559 | Pyruvate dehydrogenase E1 component subunit alpha, somatic form, mitochondrial | YGMGTsVER | -1.03 | S232 | enzymatic activity, inhibited |  |  |
| 9 | P08559 | Pyruvate dehydrogenase E1 component subunit alpha, somatic form, mitochondrial | YGmGTsVER | -2.07 | S232 | enzymatic activity, inhibited |  |  |
| 9 | P08559 | Pyruvate dehydrogenase E1 component subunit alpha, somatic form, mitochondrial | YHGHsmSDPGVsYR | -1.14 | S293;S300 | enzymatic activity, inhibited | enzymatic activity, inhibited |  |
| 9 | P31749 | RAC-alpha serine/threonine-protein kinase | SGsPSDNsGAEEMEVSLAkPk | -1.26 | S124;S129 | enzymatic activity, induced | enzymatic activity, induced | GSK2141795 |
| 9 | Q15139 | Serine/threonine-protein kinase D1 | aLGERVsIL | 1.28 | S910 | enzymatic activity, induced |  |  |
|  |  |  |  |  |  |  |  |  |
| 10 | Q9Y2I7 | 1-phosphatidylinositol-3-phosphate 5-kinase | SAsITNLSLDR | -1.39 | S307 | enzymatic activity, induced |  |  |
| 10 | P13569 | Cystic fibrosis transmembrane conductance regulator | rLsLVPDSEQGEAILPR | -4.08 | S737 | enzymatic activity, inhibited |  |  |
| 10 | Q06210 | Glucosamine--fructose-6-phosphate aminotransferase [isomerizing] 1 | vDsTTcLFPVEEk | -2.33 | S261 | enzymatic activity, induced |  |  |
| 10 | P49841 | Glycogen synthase kinase-3 beta | TTsFAESckPVQQPSAFGSMk | 1.01 | S9 | enzymatic activity, inhibited |  |  |
| 10 | P49841 | Glycogen synthase kinase-3 beta | gEPNVSyIcSR | 1.80 | Y216 | enzymatic activity, induced |  |  |
| 10 | P49841 | Glycogen synthase kinase-3 beta | qLVRGEPNVSyIcSR | 1.57 | Y216 | enzymatic activity, induced |  |  |
| 10 | Q13547 | Histone deacetylase 1 | iAcEEEFsDsEEEGEGGRk | -1.57 | S421;S423 | enzymatic activity, induced | enzymatic activity, induced | Vorinostat |
| 10 | P28482 | Mitogen-activated protein kinase 1 | vADPDHDHTGFLtEyVATR | 1.28 | T185;Y187 | enzymatic activity, induced | enzymatic activity, induced | AEZS-131 |
| 10 | Q16539 | Mitogen-activated protein kinase 14 | hTDDEMtGyVATR | 2.20 | T180;Y182 | enzymatic activity, induced | enzymatic activity, induced |  |
| 10 | P29474 | Nitric oxide synthase, endothelial | iRtQsFSLQER | -1.94 | T1175;S1177 |  | enzymatic activity, induced |  |
| 10 | P08559 | Pyruvate dehydrogenase E1 component subunit alpha, somatic form, mitochondrial | YGmGTsVER | -2.81 | S232 | enzymatic activity, inhibited |  |  |
| 10 | P08559 | Pyruvate dehydrogenase E1 component subunit alpha, somatic form, mitochondrial | YHGHsmSDPGVsYR | -1.58 | S293;S300 | enzymatic activity, inhibited | enzymatic activity, inhibited |  |
| 10 | P08559 | Pyruvate dehydrogenase E1 component subunit alpha, somatic form, mitochondrial | YHGHsMSDPGVsYR | -1.47 | S293;S300 | enzymatic activity, inhibited | enzymatic activity, inhibited |  |
| 10 | P31749 | RAC-alpha serine/threonine-protein kinase | SGsPSDNsGAEEMEVSLAkPk | 1.26 | S124;S129 | enzymatic activity, induced | enzymatic activity, induced | GSK2141795 |
| 10 | P04049 | RAF proto-oncogene serine/threonine-protein kinase | SAsEPSLHR | 1.49 | S621 | enzymatic activity, inhibited/induced |  | Sorafenib |
| 10 | P10398 | Serine/threonine-protein kinase A-Raf | SAsEPSLHR | 1.49 | S582 | enzymatic activity, induced |  | Sorafenib |
| 10 | Q15139 | Serine/threonine-protein kinase D1 | aLGERVsIL | -1.23 | S910 | enzymatic activity, induced |  |  |
|  |  |  |  |  |  |  |  |  |
| 11 | P52564 | Dual specificity mitogen-activated protein kinase kinase 6 | mcDFGISGYLVDsVAk | -2.23 | S207 | enzymatic activity, inhibited |  |  |
| 11 | P49840 | Glycogen synthase kinase-3 alpha | qLVRGEPNVSyIcSR | 1.38 | Y279 | enzymatic activity, induced |  |  |
| 11 | P28482 | Mitogen-activated protein kinase 1 | vADPDHDHTGFLtEyVATR | -1.07 | T185;Y187 | enzymatic activity, induced | enzymatic activity, induced | AEZS-131 |
| 11 | P29474 | Nitric oxide synthase, endothelial | iRtQsFSLQER | 1.03 | T1175;S1177 |  | enzymatic activity, induced |  |
| 11 | P08559 | Pyruvate dehydrogenase E1 component subunit alpha, somatic form, mitochondrial | YGMGTsVER | -2.81 | S232 | enzymatic activity, inhibited |  |  |
| 11 | P08559 | Pyruvate dehydrogenase E1 component subunit alpha, somatic form, mitochondrial | YGmGTsVER | -3.45 | S232 | enzymatic activity, inhibited |  |  |
| 11 | P04049 | RAF proto-oncogene serine/threonine-protein kinase | SAsEPSLHR | -1.71 | S621 | enzymatic activity, inhibited/induced |  | Sorafenib |
| 11 | P10398 | Serine/threonine-protein kinase A-Raf | SAsEPSLHR | -1.71 | S582 | enzymatic activity, induced |  | Sorafenib |
| 11 | Q9Y385 | Ubiquitin-conjugating enzyme E2 J1 | qIsFkAEVNSSGk | -1.42 | S184 | enzymatic activity, induced |  |  |
|  |  |  |  |  |  |  |  |  |
| 13 | P49840 | Glycogen synthase kinase-3 alpha | gEPNVSyIcSR | 1.15 | Y279 | enzymatic activity, induced |  |  |
| 13 | Q13547 | Histone deacetylase 1 | iAcEEEFsDsEEEGEGGRk | 1.66 | S421;S423 | enzymatic activity, induced | enzymatic activity, induced | Vorinostat |
| 13 | Q92769 | Histone deacetylase 2 | iAcDEEFsDsEDEGEGGRR | 1.42 | S422;S424 | enzymatic activity, inhibited | enzymatic activity, inhibited | Vorinostat |
| 13 | P08559 | Pyruvate dehydrogenase E1 component subunit alpha, somatic form, mitochondrial | YGMGTsVER | 2.82 | S232 | enzymatic activity, inhibited |  |  |
| 13 | P08559 | Pyruvate dehydrogenase E1 component subunit alpha, somatic form, mitochondrial | YHGHsmSDPGVSYR | 1.89 | S293 | enzymatic activity, inhibited |  |  |
| 13 | P08559 | Pyruvate dehydrogenase E1 component subunit alpha, somatic form, mitochondrial | YHGHSMsDPGVsYR | 1.01 | S295;S300 |  | enzymatic activity, inhibited |  |
| 13 | P31749 | RAC-alpha serine/threonine-protein kinase | SGsPSDNSGAEEMEVSLAkPk | 1.48 | S124 | enzymatic activity, induced |  | GSK2141795 |
| 13 | P04049 | RAF proto-oncogene serine/threonine-protein kinase | SAsEPSLHR | 1.91 | S621 | enzymatic activity, inhibited/induced |  | Sorafenib |
| 13 | P10398 | Serine/threonine-protein kinase A-Raf | SAsEPSLHR | 1.91 | S582 | enzymatic activity, induced |  | Sorafenib |
| 13 | P18031 | Tyrosine-protein phosphatase non-receptor type 1 | YRDVsPFDHSR | 1.38 | S50 | enzymatic activity, inhibited/induced |  |  |
|  |  |  |  |  |  |  |  |  |
| 14 | Q14432 | cGMP-inhibited 3,5-cyclic phosphodiesterase A | rTsLPcIPR | -1.42 | S312 | enzymatic activity, induced |  |  |
| 14 | P47712 | Cytosolic phospholipase A2 | qNPSRcsVsLSNVEAR | 1.18 | S727;S729 | enzymatic activity, induced |  |  |
| 14 | P52564 | Dual specificity mitogen-activated protein kinase kinase 6 | mcDFGISGYLVDsVAk | -2.78 | S207 | enzymatic activity, inhibited |  |  |
| 14 | P49840 | Glycogen synthase kinase-3 alpha | qLVRGEPNVSyIcSR | 1.08 | Y279 | enzymatic activity, induced |  |  |
| 14 | P08559 | Pyruvate dehydrogenase E1 component subunit alpha, somatic form, mitochondrial | YGMGTsVER | -3.02 | S232 | enzymatic activity, inhibited |  |  |
| 14 | P08559 | Pyruvate dehydrogenase E1 component subunit alpha, somatic form, mitochondrial | YGmGTsVER | -2.82 | S232 | enzymatic activity, inhibited |  |  |
| 14 | P08559 | Pyruvate dehydrogenase E1 component subunit alpha, somatic form, mitochondrial | YHGHsmSDPGVsYR | -1.38 | S293;S300 | enzymatic activity, inhibited | enzymatic activity, inhibited |  |
| 14 | P08559 | Pyruvate dehydrogenase E1 component subunit alpha, somatic form, mitochondrial | YHGHsMSDPGVsYR | -1.08 | S293;S300 | enzymatic activity, inhibited | enzymatic activity, inhibited |  |
| 14 | P04049 | RAF proto-oncogene serine/threonine-protein kinase | STsTPNVHMVSTTLPVDSR | -1.25 | S259 | enzymatic activity, inhibited |  | Sorafenib |
| 14 | Q9Y385 | Ubiquitin-conjugating enzyme E2 J1 | qIsFkAEVNSSGk | -1.12 | S184 | enzymatic activity, induced |  |  |
